# Synthesis and Characterization of Dihydrouracil Analogs Utilizing Biginelli Hybrids

**DOI:** 10.3390/molecules27092939

**Published:** 2022-05-04

**Authors:** Syed Nasir Abbas Bukhari, Hasan Ejaz, Mervat A. Elsherif, Nenad Janković

**Affiliations:** 1 Department of Pharmaceutical Chemistry, College of Pharmacy, Jouf University, Sakaka 72388, Saudi Arabia; 2 Department of Clinical Laboratory Sciences, College of Applied Medical Sciences, Jouf University, Sakaka 72388, Saudi Arabia; hetariq@ju.edu.sa; 3 Chemistry Department, College of Science, Jouf University, Sakaka 72388, Saudi Arabia; maelsherif@ju.edu.sa; 4 Department of Science, Institute for Information Technologies Kragujevac, University of Kragujevac, Jovana Cvijića bb, 34000 Kragujevac, Serbia

**Keywords:** dihydrouracil, Biginelli hybrid, synthesis, tetrahydropyrimidine, *m*-chloroperbenzoic acid

## Abstract

Dihydrouracil presents a crucial intermediate in the catabolism of uracil. The vital importance of uracil and its nucleoside, uridine, encourages scientists to synthesize novel dihydrouracils. In this paper, we present an innovative, fast, and effective method for the synthesis of dihydrouracils. Hence, under mild conditions, 3-chloroperbenzoic acid was used to cleave the carbon–sulfur bond of the Biginelli hybrids 5,6-dihydropyrimidin-4(3*H*)-ones. This approach led to thirteen novel dihydrouracils synthesized in moderate-to-high yields (32–99%).

## 1. Introduction

Uracil is one of the nucleobases, and it can be found in RNA and as a significant constituent of the DNA of certain bacterial viruses [1]. The well-known intermediate scaffold dihydrouracil is a key precursor in the catabolism of uracil, a critical building block of life [2]. Several methods have been reported for the preparation of dihydrouracil [3,4,5,6,7,8,9]. However, most of these methods have certain drawbacks such as complicated multi-step procedures [10,11], high energy consumption [12], and air-sensitive organometallic compounds [4]. For instance, preparing a dihydrouracil scaffold is based on the hydrolytic removal of the SCH_3_ or OCH_3_ group from the dihydropyrimidinone core, which is carried out in strongly acidic and/or basic conditions and at high temperatures [4]. Methods for the synthesis of DHU scaffolds include quite difficult reaction conditions. For instance, formic acids and hydrochloric acid [6] were used under heating conditions to synthesize 6-aryl-dihydrouracils. Considering a literature review, the most successful method for synthesis of 6-aryl-DHU was published by Pair et al. [5] 6-Phenyl-5,6-dihydrouracil (37%) was synthesized by applying formic acid, MsOH, and heated to reflux for 24 h.

**Figure 1:**
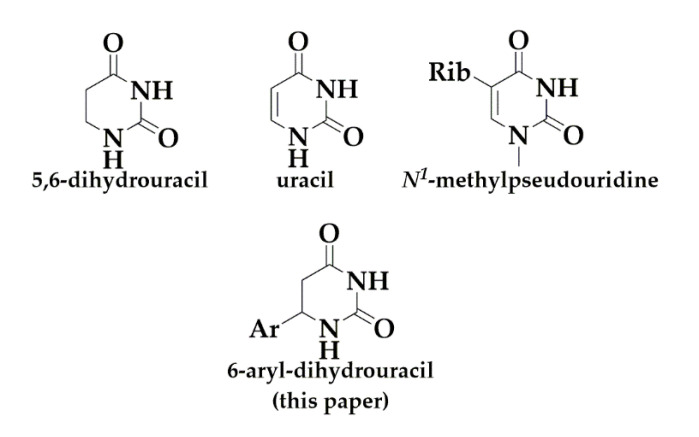
Structures of several important compounds containing the uracil motif.

Some representative uracil-based compounds are depicted in Figure 1. N^1^-methylpseudouridine is the most important uracil analog and a natural archaeal tRNA component [13,14]. Synthetic pyrimidine nucleoside is used for in vitro transcription and is also found in the SARS-CoV-2 mRNA vaccines tozinameran (Pfizer–BioNTech) and elasomeran (Moderna) [15]. Further, it is also used in vaccines against Zika [16], HIV-1 [17], and Ebola [18]. Dasabuvir, a potent non-nucleoside anti-HCV compound approved by the FDA in 2014, contains a uracil scaffold [19]. Furthermore, alkyl uracil derivatives show significant anti-HIV activity [20,21]. By considering the literature, uracil-based biological active compounds, such as 5-iodouridine [22], (*S*)-willardiine [23], and zidovudine [24], can also be found. Since 1957 to the present, the most helpful uracil molecule has been the well-known chemotherapeutic 5-fluorouracil [25]. Udayakumar et al. reported novel dihydrouracil derivatives with significant activities against A431 cancer cell lines [26]. Embrey et al. published a series of highly active DHU derivatives that contain naphthyridine scaffolds as HIV-1 integrase inhibitors at nanomolar levels [27]. The DHU analogues coupled with neomycin conjugates showed good activity against *E. coli* ATCC 25,922 and K12, even more potent compared to the control probe ciprofloxacin. In addition, few samples were more potent against *Klebsiella pneumoniae* than the control tetracycline [28]. Promising antimicrobial activity of selected N^3^-alkylated DHUs against *S. aureus* and *C. albicans* was described [29].

Considering the importance of DHU scaffolds [19], there is a reasonable trend towards development of novel routes to synthesize uracil-based compounds. In this report, Biginelli’s hybrids were chosen as starting materials for dihydrouracil (DHU) synthesis. Over the years, Biginelli chemistry has produced many valuable compounds that possess significant biological activities [30,31]. Given these facts, as well as our continual interest in heterocyclic compounds [32,33,34,35] and Biginelli chemistry [36,37,38,39,40,41], we decided to explore it to develop a novel and easier synthetic strategy for the synthesis of 6-aryl-dihydrouraciles (DHUs).

## 2. Results and Discussion

**Scheme 1:**
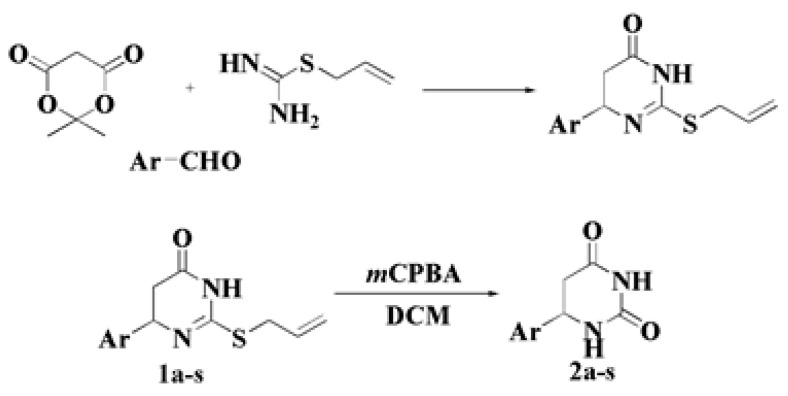
General outline of the synthesis of DHUs [41].

In this paper, a simple, fast, and efficient method for the synthesis of DHUs (**2**) from different 6-aryl-5,6-dihydropyrimidin-4(3*H*)-ones (**1a-s**) is presented (Scheme 1). The starting materials required for this methodology, **1a-s**, were synthesized as a racemic mixture following published method [41].

The first goal of this project was to optimize the reaction conditions. Compound **1a** was chosen as a model substrate for the study, and it was subjected to a variety of reagents, such as 6M HCl, sodium periodate, formic acid, and sodium hydroxide, in polar solvents (i.e., water, methanol, and THF) to synthesize **2a**. Under these reagent and solvent conditions, compound **1a** was decomposed into an unidentifiable mixture of products. However, the use of phosphotungstic acid in absolute ethanol gave DHU **2a** in lower yield (30%) after 24 h. Subsequent attempts to synthesize **2a** from **1a** were realized by applying *m*-chloroperbenzoic acid (mCPBA) as the reagent in different solvents (Table 1).

The reaction was performed in six different solvents using an excess of mCPBA (2.2 eq.), and the desired product, **2a**, was observed in five of those with DCM affording the maximum yield, 75% in 3 h (entry 5). To decipher the reasoning for the varying yields, we studied certain properties of these solvents such as the dielectric constants, polarity, and the index of the solvent dipolarity/polarizability. Water, DCM, THF, CHCl_3_, toluene, and dioxane have dielectric constants of 80, 9.1, 7.6, 4.8, 2.4, and 2.3, respectively [42,43]. It has been shown that the higher polarity of a solvent affects the yield, but this effect is not alone; the hydrogen bond acceptor (HBA) ability of the solvent and the index of solvent dipolarity/polarizability π* can also influence the reaction. The HBA parameter describes the ability of the solvent to accept a proton in a solvent-to-solute hydrogen bond. Namely, THF, dioxane, and water have HBA numbers in the following order 0.52, 0.38, and 0.14, respectively (Table 2) [44,45]. We suspect that the yield of **2a** was negatively affected by the presence of the HBA properties of water, dioxane, or THF. Toluene, DCM, and chloroform do not have HBA properties. Considering this, we proposed that the solvent molecule with HBA properties (i.e., dioxane, THF, and water) interacts with NH protons and, thus, make access of the reagent (mCPBA) to the reaction center (thioureide fragment; HN-(C-S-allyl)-N=) more difficult. This fact could be crucial for the such different (lower) yields achieved in a solvent with HBA (i.e., dioxane (32%), THF (29%), and water (-)) compared to the yield of **2a** that was noted in solvents without HBA properties (i.e., toluene (51%), DCM (75%), and chloroform (40%)). In addition, the π* scale is an index that measures the ability of the solvent to stabilize a charge or a dipole by virtue of its dielectric effect. As can be seen from Table 2, among non-HBA solvents (i.e., toluene, DCM, and CHCl_3_), chloroform has the highest π* index (Table 2). Reactions in chloroform and toluene gave **2a** in similar yields (i.e., 40% and 51%, respectively). Both solvents do not have HBA properties (HBA = 0) and have a similar π* index (chloroform = 0.58 and toluene = 0.54; Table 2). The absence of HBA properties combined with a high π* index could be the reason for the highest yield for **2a** achieved in a reaction carried out in DCM (HBA = 0 and π* index = 0.82) in comparison to toluene or chloroform.

**Figure 2:**
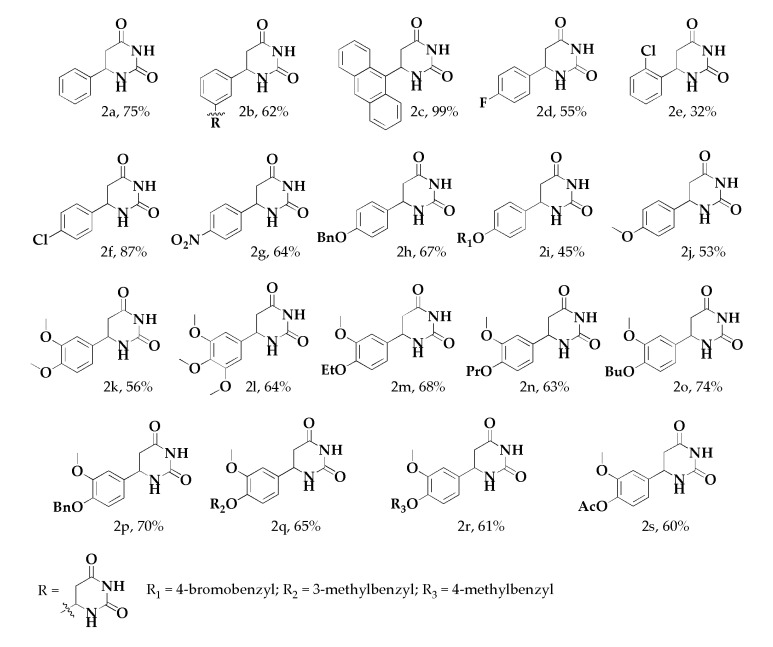
Structures and isolated yields of the DHUs.

To investigate the scope of the reaction (Scheme 1), the same reaction conditions were applied (2 mmol of 1, 2.2 eq. of mCPBA and r.t., 3 h) to a series of 6-aryl-5,6-dihydropyrimidin-4(3*H*)-ones (**1b-s**). In all these reactions, the targeted DHUs crashed out from the solution, which were isolated and characterized using NMR and IR spectroscopy after simple work up. In one case, a nearly quantitative yield was noted (**2b**, 99%). In general, the transformation afforded good-to-excellent yields of the product, except in the case of compound **1e** with an *o*-chlorophenyl substitution (**2e**, 32%). A total of 19 DHUs were prepared among which 13 were prepared for the first time. The structures and isolated yields of these derivatives are presented in Figure 2.

The applied reaction conditions showed good tolerance to the substituents on the aromatic ring. As a result, substrates with both an electron donating and an electron withdrawing group at the *para* position on the aromatic ring provided similar yields. For example, 4’-fluoro, -chloro, -nitro, and -benzyloxy gave the corresponding DHUs at 55, 87, 64 and 67%, respectively. Considering yield outcomes, the presence of alkoxy function (methoxy(**2j**), ethoxy (**2m**), butoxy (**2o**), benzyloxy (**2p**), or 3’-methylbenzyloxy (**2q**)), even acetoxy group at *para* position (**2s**) also demonstrated good group tolerance. Interestingly, however, the aryl groups phenyl or antracen-10’-yl positioned at the C4 position, even though they possess similar electron-withdrawing behavior, realized different yields of DHU (75% of **2a** and 99% of **2b**). 

**Figure 3:**
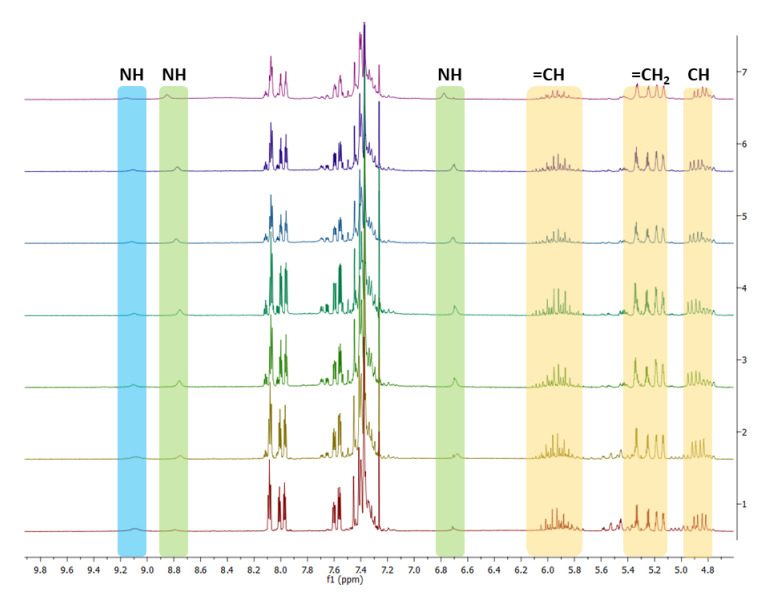
Stacked NMR spectra of **1a** and mCPBA in CDCl_3_. The blue and green shapes represent NH protons that originate from **1a** and product **2a**, respectively; the yellow shapes represent double bonds and benzylic protons.

Going forward, we followed the reaction between **1a** and mCPBA using ^1^H NMR in CDCl_3_ as a solvent (Figure 3). For this purpose, we prepared solutions of **1a** (300 μL, 120 mM) and mCPBA (300 μL, 260 mM). Immediately after mixing, the first spectra were recorded. Six NMR spectra were then recorded every twelve hours.

As seen from stacked spectra in Figure 3, the amide proton originating from **1a** nearly disappeared. The intensity of the amide protons in the products increased (**2a**, green shapes; Figure 3) and was followed by a decrease in amide proton intensity from **1a** (blue shape; Figure 3). Furthermore, mCPBA did not react with the double bond or even with the benzylic position. Double-bond protons showed the same multiplets in the range 5.1–5.4 ppm (=CH_2_) and 5.7–6.1 ppm (=CH) (Figure 3; orange shapes).

**Figure 4:**
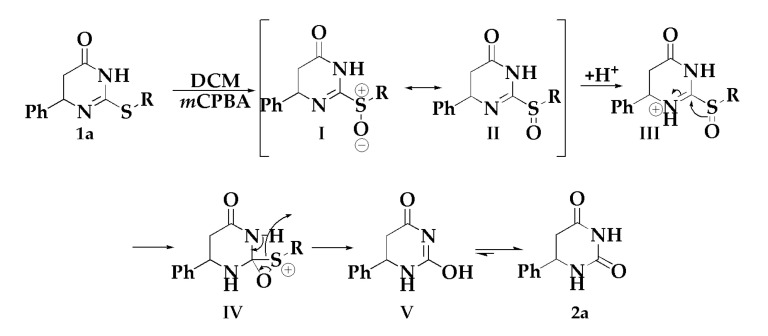
Proposed mechanism (R = allyl).

The NMR experiments provided us with valuable information: (a) the double bond did not react with peracid, even though mCPBA can easily transform the double bond into an epoxide ring; (b) benzylic protons originating from a dihydropyrimidine core is also sensitive to the presence of oxidants [39], but the applied peracid had no significant effect on the chemical shifts of the benzylic protons, implying its stability under the applied conditions. Taking into account the data obtained from the NMR investigation, we proposed a plausible mechanism for the transformation (Figure 4). In the initial step, the sulfide group is oxidized into sulfoxide (II), which upon protonation forms intermediate III. Sulfoxide oxygen in intermediate III attacks C2, forming a C–O bond followed by cleavage of the C–S bond, and elimination of thiol can then give rise to the observed product **2a**. A similar ring contraction in dihydropyrimidine compounds has already been suggested [46].

In summary, an elegant approach to novel dihydrouracils has been developed. In most syntheses, moderate-to-high yields of target compounds were realized. The advantage of our method over existing ones is that we do not use metals, strong bases, or acids and work at room temperature. In addition, a simple work up process, good yields, and broad substrate scope could also be additional benefits of the presented method. After applying uracil’s derivative (N^1^-methyl-pseudouridine) into COVID-19 vaccines, we firmly believe that uracil analogs have a bright future. Furthermore, the crucial importance of developing new approaches for the synthesis of dihydrouracil lies in the importance of these molecules, both in biological processes and in the development of new antiviral drugs.

## 3. Materials and Methods

The melting points (mp) were determined on a Mel-Temp apparatus and were uncorrected. The IR spectra were recorded using a Perkin–Elmer Spectrum One FT-IR spectrometer on a KBr pellet. The NMR spectra of compounds **2a-s** were performed in DMSO-*d*_6_ with TMS as the internal standard on a Varian Gemini 200 MHz NMR spectrometer (^1^H at 200 and ^13^C at 50 MHz). The abbreviations for the NMR signals that were used are s = singlet, d = doublet, t = triplet, m = multiplet, and br. s. = broad singlet. ^1^H and ^13^C spectra are given in the Supplementary Materials (Figures S1–S38).

Synthesis of DHUs (**2**): in a 25 mL round-bottomed flask, appropriate dihydropyrimidine **1a-s** (2 mmol) was dissolved in 5 mL of dichloromethane. Then, 2.2 eq of *m*CPBA was loaded at room temperature. The reactions were completed for 3 h. The precipitated product was filtered, washed with DCM, and dried in a vacuum. Dry powder was treated with saturated sodium bicarbonate solution, then filtrated, washed with water, and dried.

*6-Phenyl-dihydropyrimidine-2,4(1H,3H)-dione***2a**: white powder; yield: 75%; Mp = 229 °C; IR (KBr) ν 3432, 3209, 1738, 1695, and 1452 cm^−1^; ^1^H NMR (200 MHz, DMSO-*d*_6_) δ 10.18 (s, 1H, NH), 8.01 (s, 1H, NH), 7.42–7.22 (m, 5H, Ar), 4.68 (td, *J* = 6.4, 2.5 Hz, 1H, CH), and 2.73 (ddd, *J* = 23.1, 16.3, 6.3 Hz, 2H, CH_2_) ppm; ^13^C NMR (50 MHz, DMSO-*d*_6_) δ 170.0, 154.0, 141.4, 128.8, 127.8, 126.2, 50.3, and 38.4 ppm; Calcd. for C_10_H_10_N_2_O_2_: 63.15; H, 5.30; N, 14.73; Found: C 62.95, H 5.20, and N 14.62 (%).

*Dihydro-6-(3’-(hexahydro-2”,6”-dioxopyrimidin-4”-yl)phenyl)pyrimidine-2,4(1H,3H)-dione***2b**: white powder; yield: 64%; Mp = 191 °C; IR (KBr) ν 3415, 3201, 1741, 1695, 1458, and 1300 cm^−1^; ^1^H NMR (200 MHz, DMSO-*d*_6_) δ 10.19 (s, 1H, NH), 7.99 (s, 1H, NH), 7.43–7.23 (m, 2H, Ar), 4.72–4.66 (m, 1H, CH), and 2.87–2.55 (m, 2H, CH_2_) ppm; ^13^C NMR (50 MHz, DMSO-*d*_6_) δ 169.7, 153.8, 141.6, 128.9, 125.3, 124.4, 50.4, and 38.5 ppm; Calcd. for C_14_H_14_N_4_O_4_: 55.63; H, 4.67; N, 18.53; Found: C 55.47, H 4.60, N 18.45 (%).

*6-(anthracen-10′-yl)-dihydropyrimidine-2,4(1H,3H)-dione***2c** Light yellow powder; Yield: 99%; Mp = 254 °C; IR (KBr) ν 3392, 3188, 1732, 1696, 1655, 1470, 1297 cm^−1^; ^1^H NMR (200 MHz, DMSO-*d*_6_) δ 10.39 (s, 1H, NH), 8.64 (m, 3H, NH + Ar), 8.13 (m, 1H, Ar), 7.90 (m, 2H, Ar), 7.69–7.48 (m, 4H, Ar), 6.41 (dd, *J* = 13.3, 4.6 Hz, 1H, CH), 3.45–3.30 (m, 1H, CH), 2.68–2.57 (m, 1H, CH) ppm; ^13^C NMR (50 MHz, DMSO-*d*_6_) δ 170.1, 166.3, 153.9, 133.3, 132.3, 131.4, 130.5, 129.7, 129.4, 128.9, 128.7, 127.9, 126.1, 125.1, 46.5, 36.4 ppm; Before elemental analysis, **2c** was recrystallized from acetone/water mixture; calcd. for C_18_H_14_N_2_O_2_: C 74.47, H 4.86, N 9.65; found: C 74.21, H 4.75, N 9.49 (%)

*6-(4′-fluorophenyl)-dihydropyrimidine-2,4(1H,3H)-dione***2d** White powder; Yield: 55%; Mp = 260 °C; IR (KBr) ν 3205, 3085, 1738, 1695, 1516, 1443 cm^−1^; ^1^H NMR (200 MHz, DMSO-*d*_6_) δ 10.20 (s, 1H, NH), 8.01 (s, 1H, NH), 7.43–7.15 (m, 4H, Ar), 4.73–4.66 (m, 1H, CH), 2.95–2.57 (m, 2H, CH_2_) ppm; ^13^C NMR (50 MHz, DMSO-*d*_6_) δ 169.7, 164.0, 159.2, 153.8, 137.3, 137.3, 128.3, 128.2, 115.6, 115.2, 49.7, and 38.4 ppm; calcd. for C_10_H_9_FN_2_O_2_: C 57.69, H 4.36, and N 13.46; found: C 57.45, H 4.25, N 13.25 (%).

*6-(2’-Chlorophenyl)-dihydropyrimidine-2,4(1H,3H)-dione***2e**: white powder; yield: 32%; Mp = 212 °C; IR (KBr) ν 3241, 3078, 1706, 1490, and 1438 cm^−1^; ^1^H NMR (200 MHz, DMSO-*d*_6_) δ 10.35 (s, 1H, NH), 8.01 (s, 1H, NH), 7.51–7.27 (m, 4H, Ar), 4.97–4.93 (m, 1H, CH), 2.98 (dd, *J* = 16.4, 6.3 Hz, 1H), and 2.53 (dd, *J* = 16.3, 6.1 Hz, 1H) ppm; ^13^C NMR (50 MHz, DMSO-*d*_6_) δ 169.2, 153.9, 138.1, 131.3, 129.9, 129.6, 127.8, 127.1, 47.7, and 36.4 ppm; calcd. for C_10_H_9_ClN_2_O_2_: C 53.47, H 4.04, and N 15.78; found: C 53.35, H 3.95, and N 15.70 (%).

*6-(4’-Chlorophenyl)-dihydropyrimidine-2,4(1H,3H)-dione***2f**: Light yellow powder; yield: 87%; Mp = 249 °C; IR (KBr) ν 3241, 3080, 1700, and 1476 cm^−1^; ^1^H NMR (200 MHz, DMSO-*d*_6_) δ 10.21 (s, 1H, NH), 8.02 (s, 1H, NH), 7.40 (dd, *J* = 20.0, 8.4 Hz, 4H, Ar), 4.71 (m, 1H, CH), 2.89–2.78 (dd, *J* = 16.4, 5.7 Hz, 1H, CH), and 2.67–2.56 (dd, *J* = 16.3, 7.1 Hz, 1H, CH) ppm; ^13^C NMR (50 MHz, DMSO-*d*_6_) δ 169.6, 153.8, 140.2, 132.3, 128.6, 128.11, 49.7, and 38.2 ppm; calcd. for C_10_H_9_ClN_2_O_2_: C 53.47, H 4.04, and N 15.78; found: C 53.26, H 3.97, and N 15.70 (%).

*6-(4’-Nitrophenyl)-dihydropyrimidine-2,4(1H,3H)-dione***2g**: white powder; yield: 64%; Mp = 243 °C; IR (KBr) ν 3243, 3078, 1710, 1521, 1490, and 1346 cm^−1^; ^1^H NMR (200 MHz, DMSO-*d*_6_) δ 10.29 (s, 1H, NH), 8.25 (d, *J* = 8.7 Hz, 2H, Ar), 8.16 (s, 1H, NH), 7.62 (d, *J* = 8.6 Hz, 2H, Ar), 4.87 (s, 1H, CH), and 2.79 (ddd, *J* = 23.4, 16.4, 6.4 Hz, 2H, CH_2_) ppm; ^13^C NMR (50 MHz, DMSO-*d*_6_) δ 169.3, 153.7, 148.8, 147.1, 127.6, 123.8, 49.9, and 37.9 ppm; calcd. for C_10_H_9_N_3_O_4_: C 51.07, H 3.86, and N 17.87; found: C 49.90, H 3.80, and N 17.81 (%).

*6-(4’-Benzyloxyphenyl)-dihydropyrimidine-2,4(1H,3H)-dione***2h**: white powder; yield: 67%; Mp = 209 °C; IR (KBr) ν 3432, 3209, 1737, 1696, 1514, and 1452 cm^−1^; ^1^H NMR (200 MHz, DMSO-*d*_6_) δ 10.16 (s, 1H, NH), 7.95 (s, 1H, NH), 7.46–7.22 (m, 7H, Ar), 7.01 (d, *J* = 8.6 Hz, 2H, Ar), 5.10 (s, 2H, OCH_2_), 4.61 (m, 1H, CH), and 2.83–2.54 (m, 2H, CH_2_) ppm; ^13^C NMR (50 MHz, DMSO-*d*_6_) δ 169.9, 157.9, 153.8, 137.1, 133.3, 128.5, 127.8, 127.6, 127.4, 114.9, 69.4, 49.8, and 38.5 ppm; calcd. for C_17_H_16_N_2_O_3_: C 68.91, H 5.44, and N 9.45; found: C 68.75, H 5.32, and N 9.40 (%).

*6-(4’-(4”-Bromobenzyloxy)phenyl)-dihydropyrimidine-2,4(1H,3H)-dione***2i**: white powder; yield: 45%; Mp = 246 °C; IR (KBr) ν 3206, 3089, 1739, 1696, 1514, and 1456 cm^−1^; ^1^H NMR (200 MHz, DMSO-*d*_6_) δ 10.16 (s, 1H, NH), 7.95 (s, 1H, NH), 7.59 (d, *J* = 8.3 Hz, 2H, Ar), 7.40 (d, *J* = 8.3 Hz, 2H, Ar), 7.25 (d, *J* = 8.6 Hz, 2H, Ar), 7.00 (d, *J* = 8.6 Hz, 2H, Ar), 5.08 (s, 2H, OCH_2_), 4.61 (s, 1H, CH), and 2.83–2.54 (m, 2H, CH_2_) ppm; ^13^C NMR (50 MHz, DMSO-*d*_6_) δ 169.9, 157.63, 153.8, 136.6, 133.5, 131.4, 129.8, 127.4, 121.0, 115.0, 68.6, 49.7, and 38.5 ppm; calcd. for C_17_H_15_BrN_2_O_3_: C 54.42, H 4.03, and N 7.47; found: C 54.10, H 3.95, and N 7.35 (%).

*Dihydro-6-(4’-methoxyphenyl)pyrimidine-2,4(1H,3H)-dione***2j**: white powder; yield: 53%; Mp = 220 °C; IR (KBr) ν 3256, 1731, 1692, and 1510, cm^−1^; ^1^H NMR (200 MHz, DMSO-*d*_6_) δ 10.14 (s, 1H, NH), 7.94 (s, 1H, NH), 7.24 (m, 2H, Ar), 6.93 (m, 2H, Ar), 4.65–4.57 (m, 1H, CH), 3.74 (s, 3H, OCH_3_), and 2.83–2.57 (m, 2H, CH_2_); ^13^C NMR (50 MHz, DMSO-*d*_6_) δ 169.9, 158.8, 153.8, 133.1, 127.33, 114.1, 55.3, 49.7, and 38.5 ppm; calcd. for C_11_H_12_N_2_O_3_: C 59.99, H 5.49, and N 12.72; found: C 59.81, H 5.39, and N 12.65 (%).

*6-(3’,4’-dimethoxyphenyl)-dihydropyrimidine-2,4(1H,3H)-dione***2k**: white powder; yield: 56%; Mp = 233 °C; IR (KBr) ν 3292, 3230, 1725, 1700, 1521, and 1462 cm^−1^; ^1^H NMR (200 MHz, DMSO-*d*_6_) δ 10.15 (s, 1H, NH), 7.93 (s, 1H, NH), 6.95–6.79 (m, 3H, Ar), 4.63–4.57 (m, 1H, CH), 3.74 (m, 6H, 2 × OCH_3_), and 2.82–2.59 (m, 2H, CH_2_) ppm; ^13^C NMR (50 MHz, DMSO-*d*_6_) δ 170.0, 153.9, 148.9, 148.4, 133.4, 118.0, 111.8, 110.4, 55.7, 55.7, 50.1, and 38.5 ppm; calcd. for C_12_H_14_N_2_O_4_: C 57.59, H 5.64, and N 11.19; found: C 57.34, H 5.52, and N 11.05 (%).

*Dihydro-6-(3’,4’,5’-trimethoxyphenyl)pyrimidine-2,4(1H,3H)-dione***2l**: white powder; yield: 64%; Mp = 210 °C; IR (KBr) ν 3292, 3230, 1725, 1700, 1521, and 1462 cm^−1^; ^1^H NMR (200 MHz, DMSO-*d*_6_) δ 10.17 (s, 1H, NH), 7.93 (s, 1H, NH), 6.66 (s, 2H, Ar), 4.64–4.58 (m, 1H, CH), 3.77 (s, 6H, 2 × OCH_3_), 3.64 (s, 3H, OCH_3_), and 2.82–2.61 (m, 2H, CH_2_) ppm; ^13^C NMR (50 MHz, DMSO-*d*_6_) δ 169.9, 153.9, 152.9, 136.6, 103.8, 60.1, 56.1, 50.7, and 38.5 ppm; calcd. for C_13_H_16_N_2_O_5_: C 55.71, H 5.75, and N 9.99; found: C 55.59, H 5.65, and N 9.82 (%).

*6-(4’-Ethoxy-3’-methoxyphenyl)-dihydropyrimidine-2,4(1H,3H)-dione***2m**: white powder; yield: 68%; Mp = 221 °C; IR (KBr) ν 3233, 3078, 1699, 1523, 1478, and 1236 cm^−1^; ^1^H NMR (200 MHz, DMSO-*d*_6_) δ 10.15 (s, 1H, NH), 7.93 (s, 1H, NH), 6.95–6.77 (m, 3H, Ar), 4.59 (s, 1H, CH), 3.98 (q, *J* = 6.8 Hz, 2H, OCH_2_), 3.75 (s, 3H, OCH_3_), 2.82–2.59 (m, 2H, CH_2_), and 1.31 (t, *J* = 6.9 Hz, 3H, CH_3_) ppm; ^13^C NMR (50 MHz, DMSO-*d*_6_) δ 170.0, 153.8, 149.1, 147.6, 133.4, 118.0, 113.0, 110.5, 63.9, 55.6, 50.1, 38.5, and 14.9 ppm; calcd. for C_13_H_16_N_2_O_4_: C 59.08, H 6.10, and N 10.60; found: C 58.84, H 5.95, N 10.52 (%).

*6-(4’-Propoxy-3’-methoxyphenyl)-dihydropyrimidine-2,4(1H,3H)-dione***2n**: yellow powder; yield: 63%; Mp = 205 °C; IR (KBr) ν 3220, 3095, 1717, 1685, and 1520 cm^−1^; ^1^H NMR (200 MHz, DMSO-*d*_6_) δ 10.15 (s, 1H, NH), 7.93 (s, 1H, NH), 6.92 (m, 2H, Ar), 6.79 (m, 1H, Ar), 4.59 (t, *J* = 6.4 Hz, 1H, CH), 3.88 (t, *J* = 6.6 Hz, 2H, OCH_2_), 3.76 (s, 3H, OCH_3_), 2.82–2.62 (m, 2H, CH_2_), 1.79–1.62 (m, 2H, CH_2_), and 0.96 (t, *J* = 7.4 Hz, 3H, CH_3_) ppm; ^13^C NMR (50 MHz, DMSO-*d*_6_) δ 169.9, 153.8, 149.2, 147.8, 133.5, 118.1, 113.2, 110.7, 70.0, 55.8, 50.1, 38.5, 22.3, and 10.6 ppm; calcd. for C_14_H_18_N_2_O_4_: C 60.42, H 6.52, and N 10.07; found: C 60.25, H 6.45, and N 10.01 (%).

*6-(4’-Butoxy-3’-methoxyphenyl)-dihydropyrimidine-2,4(1H,3H)-dione***2o**: white powder; yield: 74%; Mp = 195 °C; IR (KBr) ν 3231, 3077, 1718, 1688, 1522, and 1476 cm^−1^; ^1^H NMR (200 MHz, DMSO-*d*_6_) δ 10.14 (s, 1H, NH), 7.92 (s, 1H, NH), 6.95–6.76 (m, 3H, Ar), 4.59 (t, *J* = 5.3 Hz, 1H), 3.92 (t, *J* = 6.4 Hz, 2H, OCH_2_), 3.75 (s, 3H, OCH_3_), 2.82–2.58 (m, 2H, CH_2_), 1.75–1.61 (m, 2H, CH_2_), 1.51–1.32 (m, 2H, CH_2_), and 0.92 (t, *J* = 7.3 Hz, 3H, CH_3_) ppm; ^13^C NMR (50 MHz, DMSO-*d*_6_) δ 169.9, 153.8, 149.2, 147.8, 133.4, 118.1, 113.1, 110.6, 68.1, 55.7, 50.1, 38.5, 31.0, 18.9, and 13.8 ppm; calcd. for C_15_H_20_N_2_O_4_: C 61.63, H 6.90, and N 9.58; found: C 61.45, H 6.80, and N 9.51 (%).

*6-(4’-Benzyloxy-3’-methoxyphenyl)-dihydropyrimidine-2,4(1H,3H)-dione***2p**: white powder; yield: 70%; Mp = 207 °C; IR (KBr) ν 3434, 3210, 1919, 1693, 1517, and 1451 cm^−1^; ^1^H NMR (200 MHz, DMSO-*d*_6_) δ 10.14 (s, 1H, NH), 7.92 (s, 1H, NH), 7.42–7.22 (m, 5H, Ar), 7.01 (d, *J* = 8.5 Hz, 2H, Ar), 6.82 (d, *J* = 9.6 Hz, 1H, Ar), 5.07 (s, 2H, OCH_2_), 4.64–4.57 (m, 1H, CH), 3.77 (s, 3H, OCH_3_), and 2.82–2.59 (m, 2H, CH_2_) ppm; ^13^C NMR (50 MHz, DMSO-*d*_6_) δ 170.0, 153.9, 149.2, 147.3, 137.2, 133.8, 128.5, 127.9, 127.8, 118.0, 113.5, 110.6, 70.1, 55.7, 50.1, and 38.4 ppm; calcd. for C_18_H_18_N_2_O_4_: C 66.25, H 5.56, and N 8.58; found: C 66.15, H 5.47, and N 8.44 (%).

*6-(4’-(3”-Methylbenzyloxy)-3’-methoxyphenyl)-dihydropyrimidine-2,4(1H,3H)-dione***2q**: white powder; yield: 65%; Mp = 205 °C; IR (KBr) ν 3408, 3213, 1730, 1698, 1517, and 1460 cm^−1^; ^1^H NMR (200 MHz, DMSO-*d*_6_) δ 10.15 (s, 1H, NH), 7.93 (s, 1H, NH), 7.31–7.15 (m, 4H, Ar), 7.12–6.98 (m, 2H, Ar), 6.82–6.77 (m, 1H, Ar), 5.02 (s, 2H, OCH_2_), 4.60 (t, *J* = 5.5 Hz, 1H, CH), 3.77 (s, 3H, OCH_3_), 2.82–2.59 (m, 2H, CH_2_), and 2.31 (s, 3H, CH_3_) ppm; ^13^C NMR (50 MHz, DMSO-*d*_6_) δ 169.9, 153.8, 149.3, 147.4, 137.5, 137.1, 133.8, 128.5, 128.3, 124.9, 118.0, 113.6, 110.6, 70.2, 55.7, 50.1, 38.5, and 21.1 ppm; calcd. for C_19_H_20_N_2_O_4_: C 67.05, H 5.92, and N 8.23; found: C 66.90, H 5.85, and N 8.14 (%).

*6-(4’-(4”-Methylbenzyloxy)-3’-methoxyphenyl)-dihydropyrimidine-2,4(1H,3H)-dione***2r**: white powder; yield: 61%; Mp = 209 °C; IR (KBr) ν 3395, 3201, 1729, 1695, and 1515 cm^−1^; ^1^H NMR (200 MHz, DMSO-*d*_6_) δ 10.15 (s, 1H, NH), 7.94 (s, 1H, NH), 7.31 (d, 2H, Ar), 7.18 (d, 2H, Ar), 7.01–6.97 (m, 2H, Ar), 6.81–6.76 (d, 1H, Ar), 5.01 (s, 2H, OCH_2_), 4.62–4.56 (m, 1H, CH), 3.76 (s, 3H, OCH_3_), 2.81–2.59 (m, 2H, CH_2_), and 2.30 (s, 3H, CH_3_) ppm; ^13^C NMR (50 MHz, DMSO-*d*_6_) δ 169.9, 153.8, 149.3, 147.4, 137.5, 134.2, 133.8, 132.5, 128.7, 127.8, 118.0, 113.6, 110.7, 70.0, 55.8, 50.1, 38.5, and 20.9 ppm; calcd. for C_19_H_20_N_2_O_4_: C 67.05, H 5.92, and N 8.23; found: C 66.91, H 5.85, and N 8.19 (%).

*6-(4’-Acetoxy-3’-methoxyphenyl)-dihydropyrimidine-2,4(1H,3H)-dione***2s**: white powder; yield: 60%; Mp = 239 °C; IR (KBr) ν 3429, 3288, 1767, 1716, 1696, 1679, and 1453 cm^−1^; ^1^H NMR (200 MHz, DMSO-*d*_6_) δ 10.20 (s, 1H, NH), 8.00 (s, 1H, NH), 7.12–7.06 (m, 2H, Ar), 6.89 (d, *J* = 8.1 Hz, 1H, Ar), 4.68 (t, *J* = 5.6 Hz, 1H, CH), 3.77 (s, 3H, OCH_3_), 2.87–2.63 (m, 2H, CH_2_), and 2.25 (s, 3H, CH_3_CO) ppm; ^13^C NMR (50 MHz, DMSO-*d*_6_) δ 169.8, 168.5, 153.8, 150.9, 139.9, 138.8, 122.8, 118.0, 111.1, 55.9, 50.3, 38.4, and 20.5 ppm; calcd. for C_13_H_14_N_2_O_5_: C 56.11, H 5.07, and N 10.07; found: C 55.91, H 4.89, and N 10.12 (%).

## Figures and Tables

**Table 1:** Screening and optimization reaction conditions.

Entry	Conditions	Yields of 2a (%)
1	*m*CPBA/toluene	51
2	*m*CPBA/dioxane	32
3	*m*CPBA/water	-
4	*m*CPBA/CHCl_3_	40
5	*m*CPBA/DCM	75
6	*m*CPBA/THF ^a^	29

^a^ Anhydrous THF; reaction time 24 h.

**Table 2:** Solvent parameters (ε—dielectric constant; HBA—hydrogen bond acceptor number; π*—index of solvent dipolarity/polarizability) [45].

Solvent	Solvent Parameter		
	**ε**	**HBA**	π*
Toluene	2.4	-	0.54
DCM	9.1	-	0.82
CHCl_3_	4.8	-	0.58
Dioxane	2.3	0.37	0.55
Water	80	0.18	1.09
THF	7.6	0.55	0.58

## Data Availability

Not applicable.

## Supplementary Materials

The following supporting information can be downloaded at: https://www.mdpi.com/article/10.3390/molecules27092939/s1, Figures S1–S38: NMR spectra.
