# Supplementary material for: Synthesis and Characterization of Dihydrouracil Analogs Utilizing Biginelli Hybrids
Source: Molecules. 2022 May 4;27(9):2939. doi: 10.3390/molecules27092939 (PMC9099458; doi:10.3390/molecules27092939)
Supplement: Supplementary file 1 [file molecules-27-02939-s001.zip › molecules-1677281-supplementary.pdf]

MOLECULES

Supplementary Information associated with the paper

Synthesis and characterization of dihydrouracil analogs utilizing Biginelli hybrids

Syed Nasir Abbas Bukhari,[1*] Hasan Ejaz,[2] Mervat Abdelaziz Mohamed Elsherif,[3] Nenad Janković,[4*]

[1]Department of Pharmaceutical Chemistry, College of Pharmacy, Jouf University, Sakaka, Al Jouf, 72388, Saudi Arabia

[2]Department of Clinical Laboratory Sciences, College of Applied Medical Sciences, Sakaka, Jouf University, Al Jouf, 72388, Saudi Arabia

[3]Chemistry Department, College of Science, Jouf University, Al Jouf, 72388, Sakaka, Saudi Arabia

[4]University of Kragujevac, Institute for Information of Technologies Kragujevac, Department of Science, Jovana Cvijića bb, 34000 Kragujevac, Serbia

Corresponding author's e-mail address: sbukhari@ju.edu.sa and nenad.jankovic@kg.ac.rs

Table of Contents

NMR spectra of **2a-n**

*^{1}H NMR spectrum of 6-phenyl-dihydropyrimidine-2,4(1H,3H)-dione **2a*** **4**

*^{13}C NMR spectrum of 6-phenyl-dihydropyrimidine-2,4(1H,3H)-dione **2a*** **5**

*^{1}H NMR spectrum of dihydro-6-(3'-(hexahydro-2'',6''-dioxopyrimidin-4''-yl)phenyl)pyrimidine-2,4(1H,3H)-dione **2b*** **6**

*^{13}C NMR spectrum of dihydro-6-(3'-(hexahydro-2'',6''-dioxopyrimidin-4''-yl)phenyl)pyrimidine-2,4(1H,3H)-dione **2b*** **7**

*^{1}H NMR spectrum of 6-(anthracen-10'-yl)-dihydropyrimidine-2,4(1H,3H)-dione **2c*** **8**

*^{13}C NMR spectrum of 6-(anthracen-10'-yl)-dihydropyrimidine-2,4(1H,3H)-dione **2c*** **9**

*^{1}H NMR spectrum of 6-(4'-fluorophenyl)-dihydropyrimidine-2,4(1H,3H)-dione **2d*** **10**

*^{13}C NMR spectrum of 6-(4'-fluorophenyl)-dihydropyrimidine-2,4(1H,3H)-dione **2d*** **11**

*^{1}H NMR spectrum of 6-(2'-chlorophenyl)-dihydropyrimidine-2,4(1H,3H)-dione **2e*** **12**

*^{13}C NMR spectrum of 6-(2'-chlorophenyl)-dihydropyrimidine-2,4(1H,3H)-dione **2e*** **13**

*^{1}H NMR spectrum of 6-(4'-chlorophenyl)-dihydropyrimidine-2,4(1H,3H)-dione **2f*** **14**

*^{13}C NMR spectrum of 6-(4'-chlorophenyl)-dihydropyrimidine-2,4(1H,3H)-dione **2f*** **15**

*^{1}H NMR spectrum of 6-(4'-nitrophenyl)-dihydropyrimidine-2,4(1H,3H)-dione **2g*** **16**

*^{13}C NMR spectrum of 6-(4'-nitrophenyl)-dihydropyrimidine-2,4(1H,3H)-dione **2g*** **17**

*^{1}H NMR spectrum of 6-(4'-benzyloxyphenyl)-dihydropyrimidine-2,4(1H,3H)-dione **2h*** **18**

*^{13}C NMR spectrum of 6-(4'-benzyloxyphenyl)-dihydropyrimidine-2,4(1H,3H)-dione **2h*** **19**

*^{1}H NMR spectrum of 6-(4'-(4''-bromobenzyloxy)phenyl)-dihydropyrimidine-2,4(1H,3H)-dione **2i*** **20**

*^{13}C NMR spectrum of 6-(4'-(4''-bromobenzyloxy)phenyl)-dihydropyrimidine-2,4(1H,3H)-dione **2i*** **21**

*^{1}H NMR spectrum of dihydro-6-(4'-methoxyphenyl)pyrimidine-2,4(1H,3H)-dione **2j*** **22**

*^{13}C NMR spectrum of dihydro-6-(4'-methoxyphenyl)pyrimidine-2,4(1H,3H)-dione **2j*** **23**

*^{1}H NMR spectrum of 6-(3',4'-dimethoxyphenyl)-dihydropyrimidine-2,4(1H,3H)-dione **2k*** **24**

*^{13}C NMR spectrum of 6-(3',4'-dimethoxyphenyl)-dihydropyrimidine-2,4(1H,3H)-dione **2k*** **25**

^{1}H NMR spectrum of dihydro-6-(3',4',5'-trimethoxyphenyl)pyrimidine-2,4(1H,3H)-dione ***2l*** ***26***

^{13}C NMR spectrum of dihydro-6-(3',4',5'-trimethoxyphenyl)pyrimidine-2,4(1H,3H)-dione ***2l*** ***27***

^{1}H NMR spectrum of 6-(4'-ethoxy-3'-methoxyphenyl)-dihydropyrimidine-2,4(1H,3H)-dione ***2m*** ***28***

^{13}C NMR spectrum of 6-(4'-ethoxy-3'-methoxyphenyl)-dihydropyrimidine-2,4(1H,3H)-dione ***2m*** ***29***

^{1}H NMR spectrum of 6-(4'-propoxy-3'-methoxyphenyl)-dihydropyrimidine-2,4(1H,3H)-dione ***2n*** ***30***

^{13}C NMR spectrum of 6-(4'-propoxy-3'-methoxyphenyl)-dihydropyrimidine-2,4(1H,3H)-dione ***2n*** ***31***

^{1}H NMR spectrum of 6-(4'-butoxy-3'-methoxyphenyl)-dihydropyrimidine-2,4(1H,3H)-dione ***2o*** ***32***

^{13}C NMR spectrum of 6-(4'-butoxy-3'-methoxyphenyl)-dihydropyrimidine-2,4(1H,3H)-dione ***2o*** ***33***

^{1}H NMR spectrum of 6-(4'-benzyloxy-3'-methoxyphenyl)-dihydropyrimidine-2,4(1H,3H)-dione ***2p*** ***34***

^{13}C NMR spectrum of 6-(4'-benzyloxy-3'-methoxyphenyl)-dihydropyrimidine-2,4(1H,3H)-dione ***2p*** ***35***

^{1}H NMR spectrum of 6-(4'-(3''-methylbenzyloxy)-3'-methoxyphenyl)-dihydropyrimidine-2,4(1H,3H)-dione ***2q*** ***36***

^{13}C NMR spectrum of 6-(4'-(3''-methylbenzyloxy)-3'-methoxyphenyl)-dihydropyrimidine-2,4(1H,3H)-dione ***2q*** ***37***

^{1}H NMR spectrum of 6-(4'-(4''-methylbenzyloxy)-3'-methoxyphenyl)-dihydropyrimidine-2,4(1H,3H)-dione ***2r*** ***38***

^{13}C NMR spectrum of 6-(4'-(4''-methylbenzyloxy)-3'-methoxyphenyl)-dihydropyrimidine-2,4(1H,3H)-dione ***2r*** ***39***

^{1}H NMR spectrum of 6-(4'-acetoxy-3'-methoxyphenyl)-dihydropyrimidine-2,4(1H,3H)-dione ***2s*** ***40***

^{13}C NMR spectrum of 6-(4'-acetoxy-3'-methoxyphenyl)-dihydropyrimidine-2,4(1H,3H)-dione ***2s*** ***41***

6-phenyl-dihydropyrimidine-2,4(1*H*,3*H*)-dione **2a**

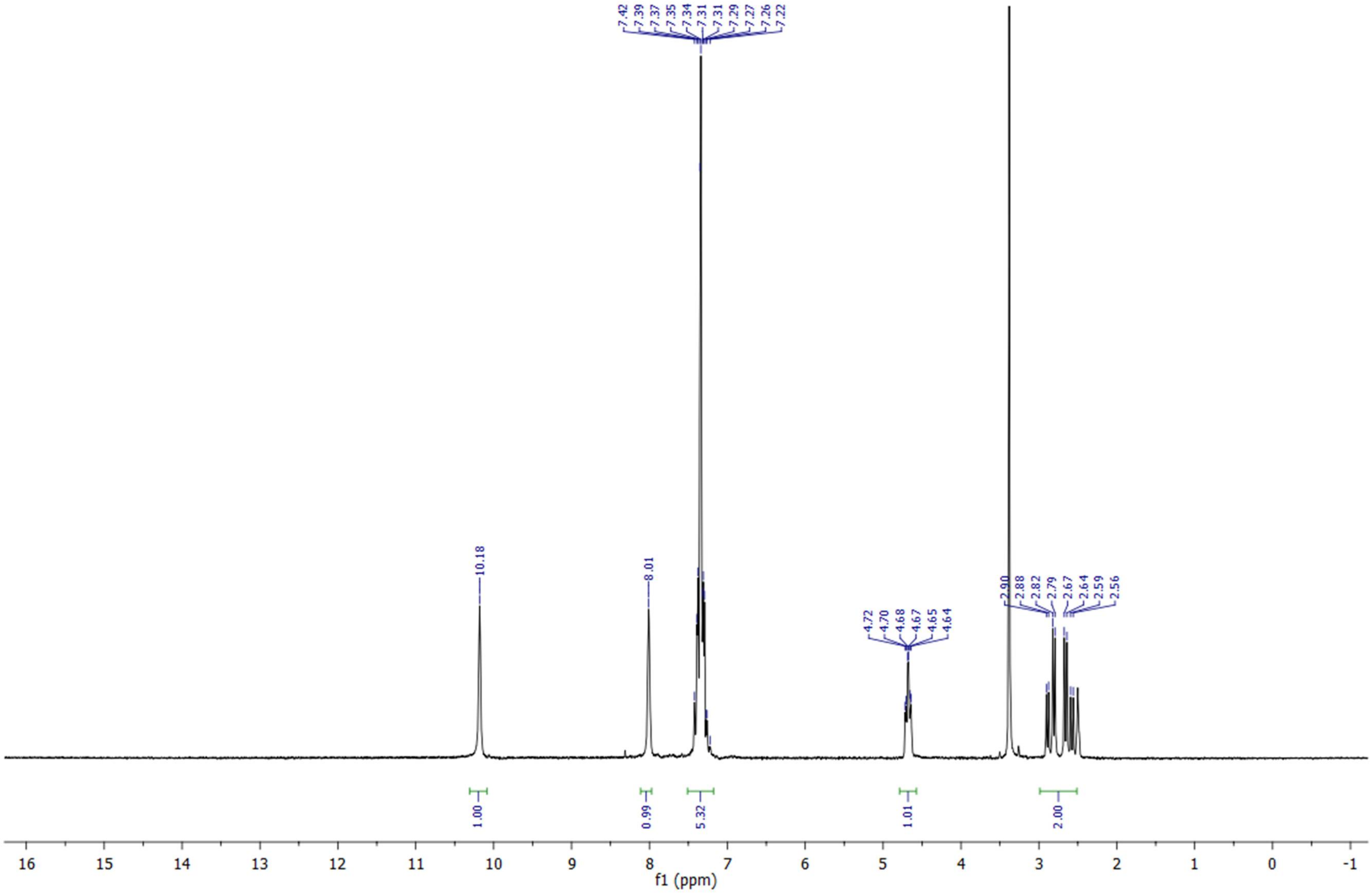

Figure S1. ^{1}H NMR spectrum of **2a**

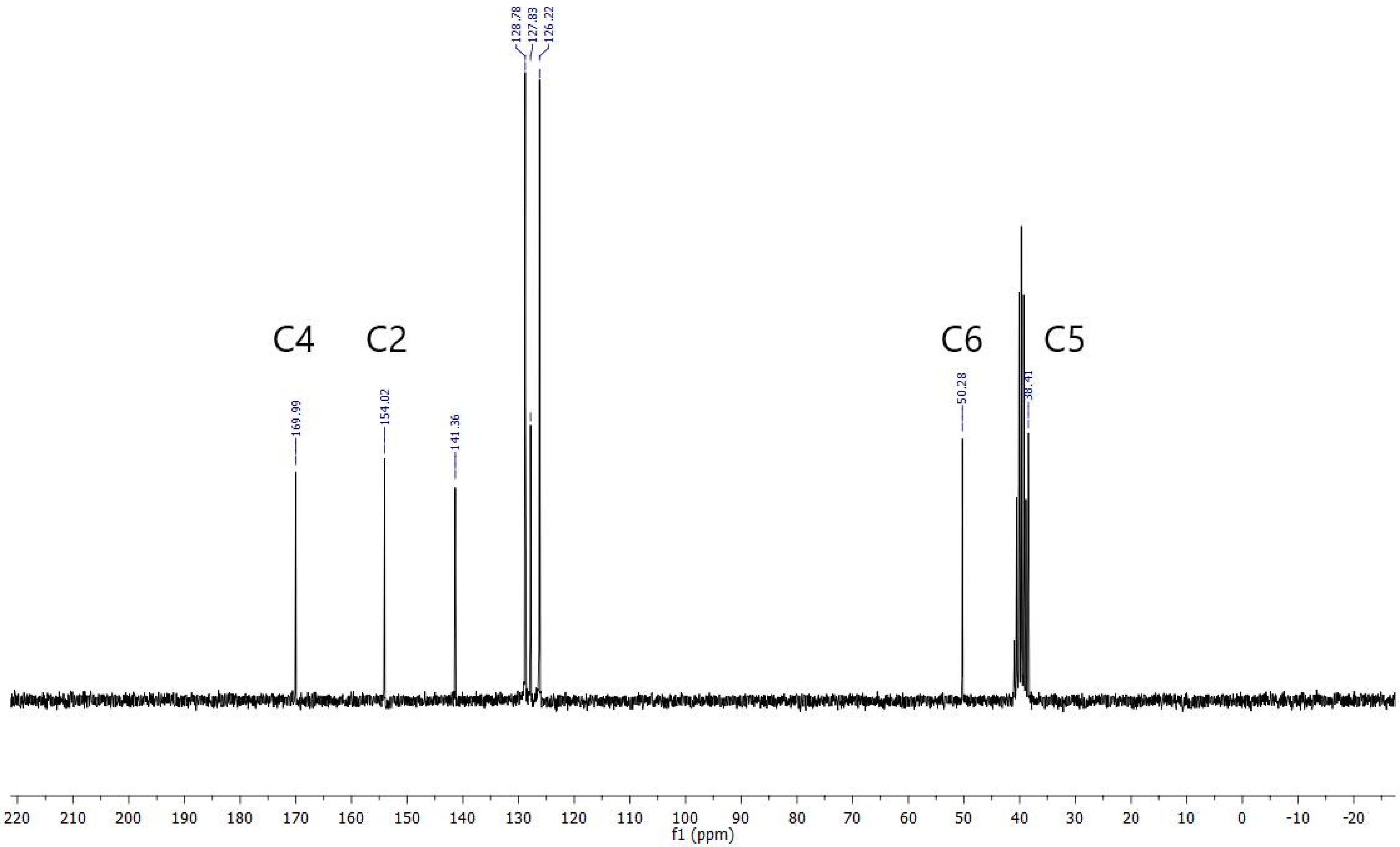

Figure S2. ^{13}C NMR spectrum of **2a**

dihydro-6-(3'-(hexahydro-2'',6''-dioxopyrimidin-4''-yl)phenyl)pyrimidine-2,4(1H,3H)-dione **2b**

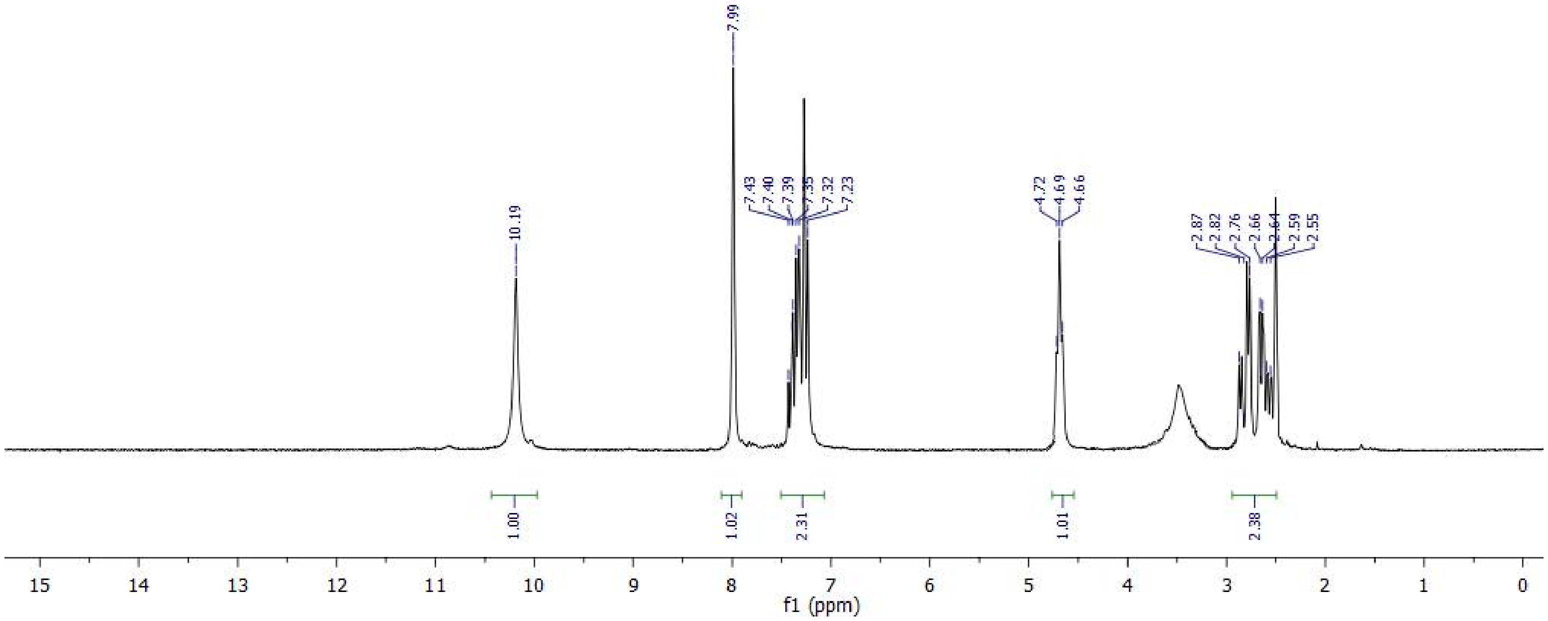

Figure S3. ^{1}H NMR spectrum of **2b**

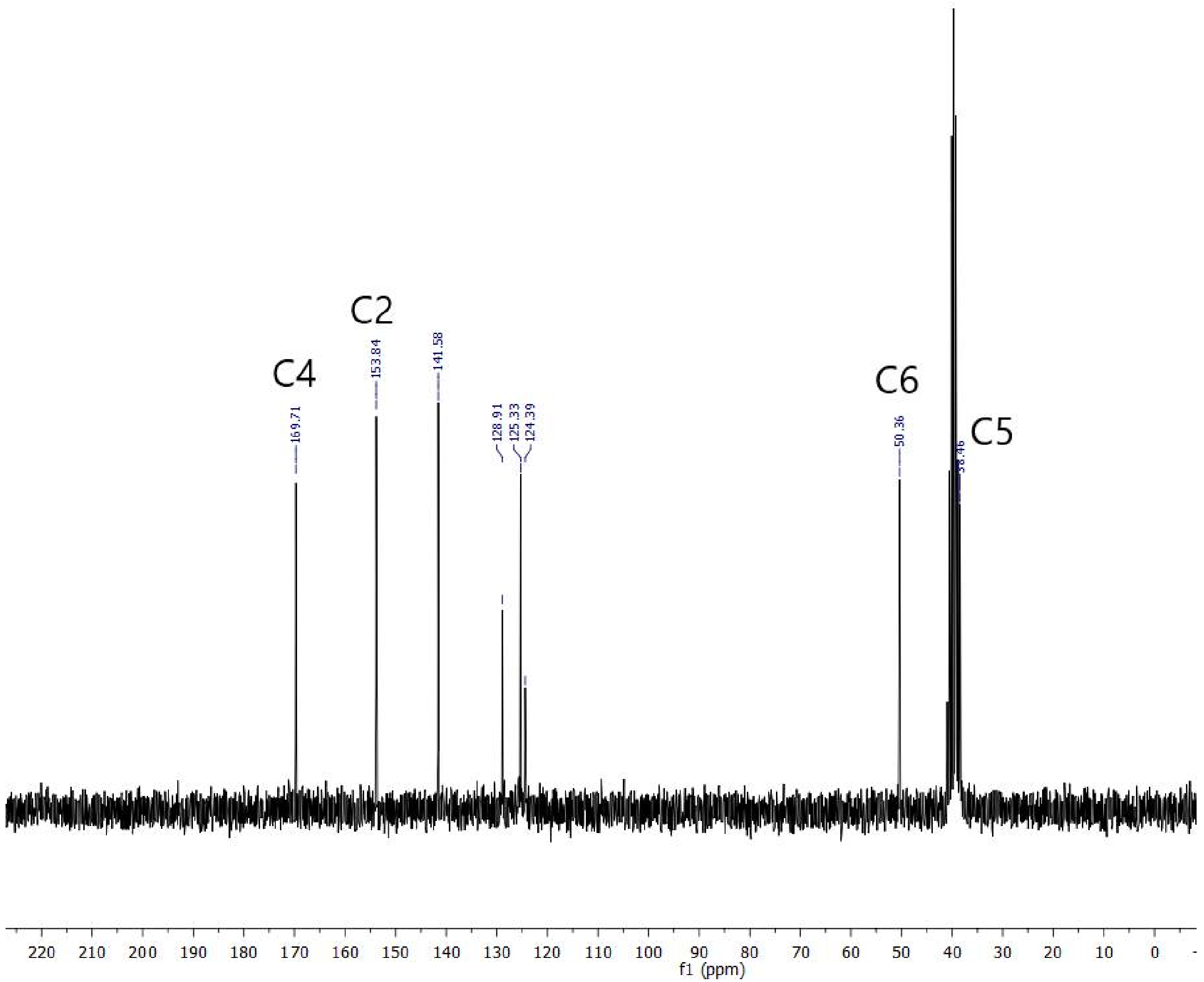

Figure S4. ^{13}C NMR spectrum of **2b**

6-(anthracen-10'-yl)-dihydropyrimidine-2,4(1*H*,3*H*)-dione **2c**

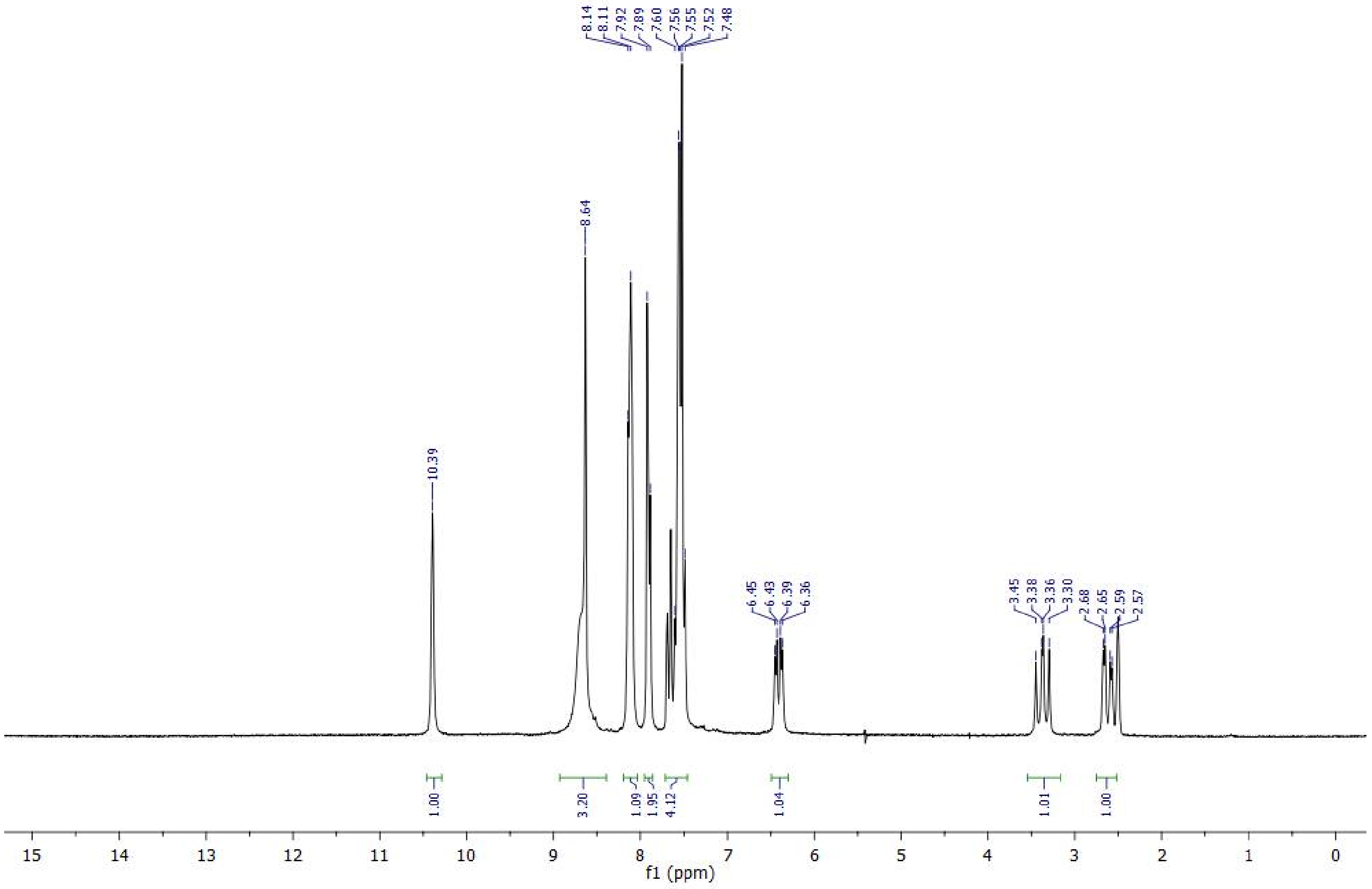

Figure S5. ^{1}H NMR spectrum of **2c** (aromatic protons in range 8.4-8.9 ppm originated from m-chlorobenzoic acid)

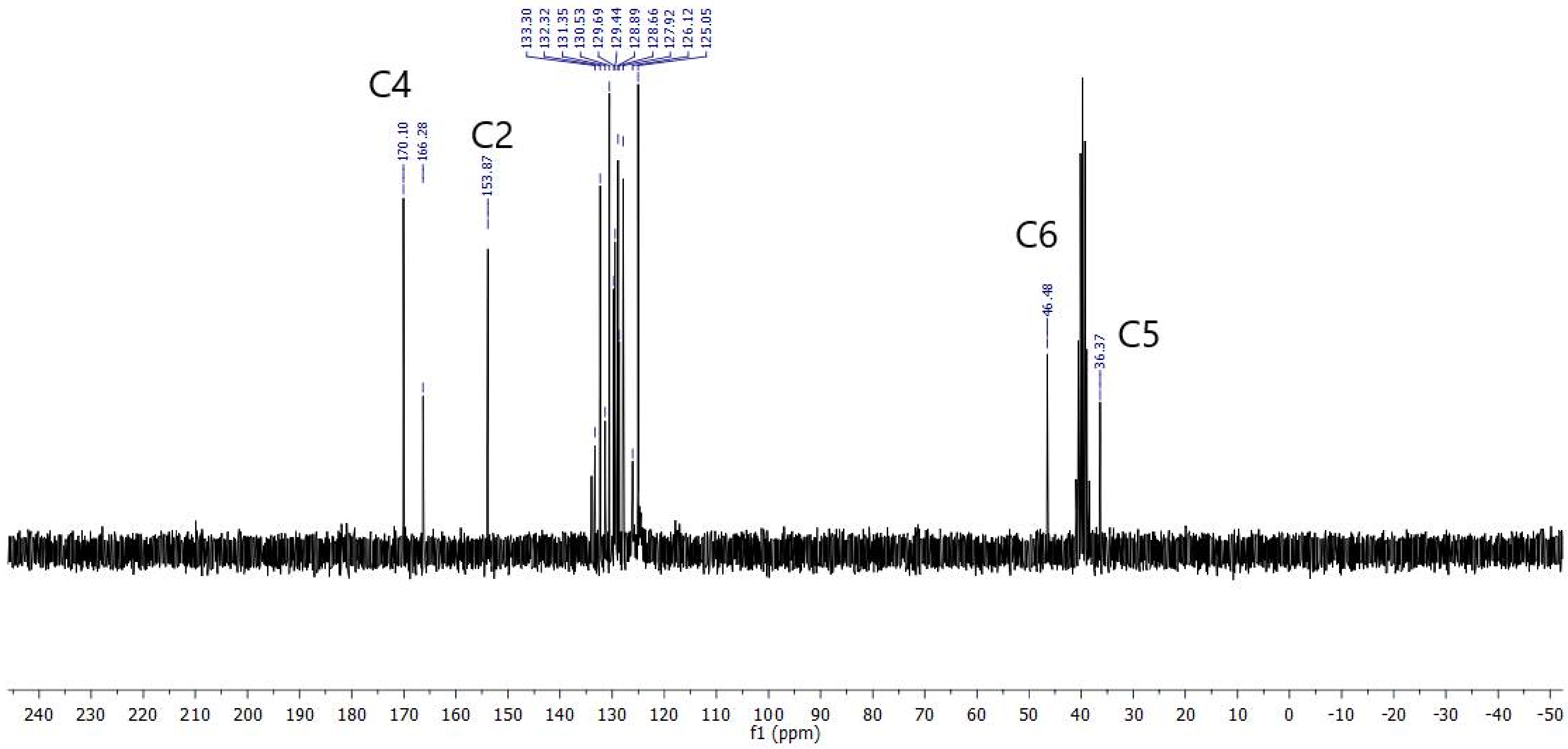

Figure S6. ^{13}C NMR spectrum of **2c** [(carbonyl (COOH) at 166.3 ppm originated from m-chlorobenzoic acid)]

6-(4’-fluorophenyl)-dihydropyrimidine-2,4(1*H*,3*H*)-dione **2d**

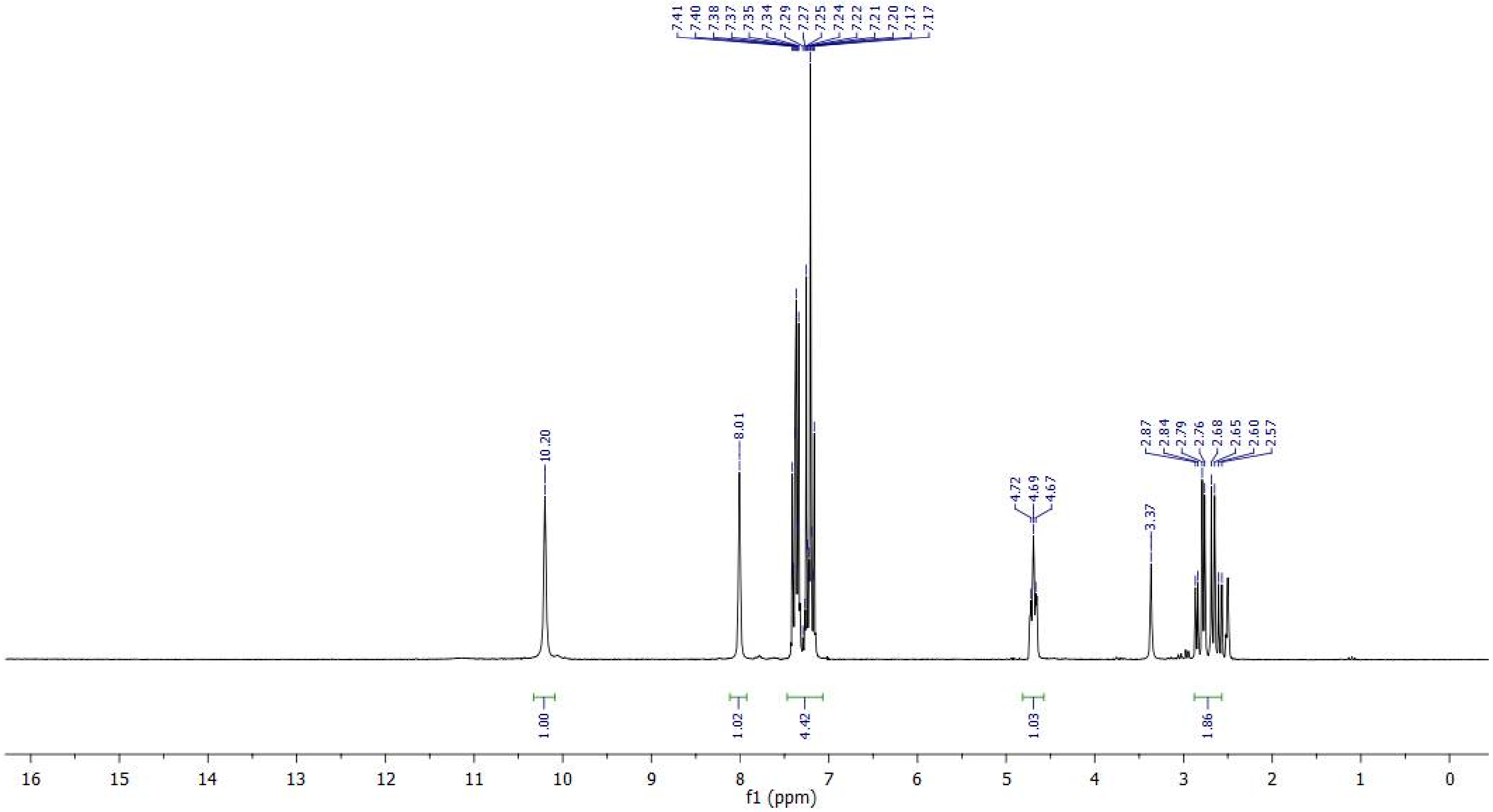

Figure S7. ^{1}H NMR spectrum of **2d**

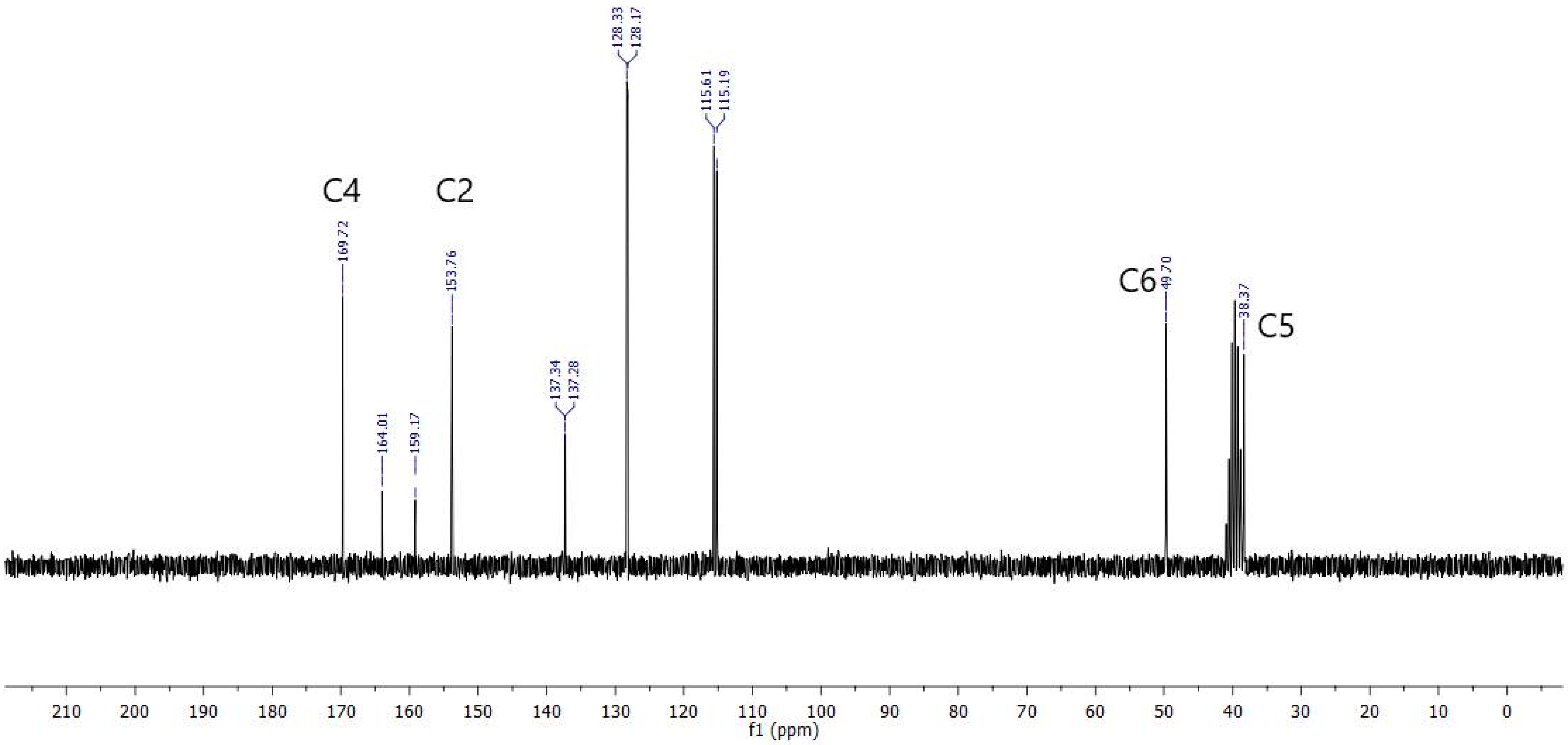

Figure S8. ^{13}C NMR spectrum of **2d**

6-(2’-chlorophenyl)-dihydropyrimidine-2,4(1*H*,3*H*)-dione **2e**

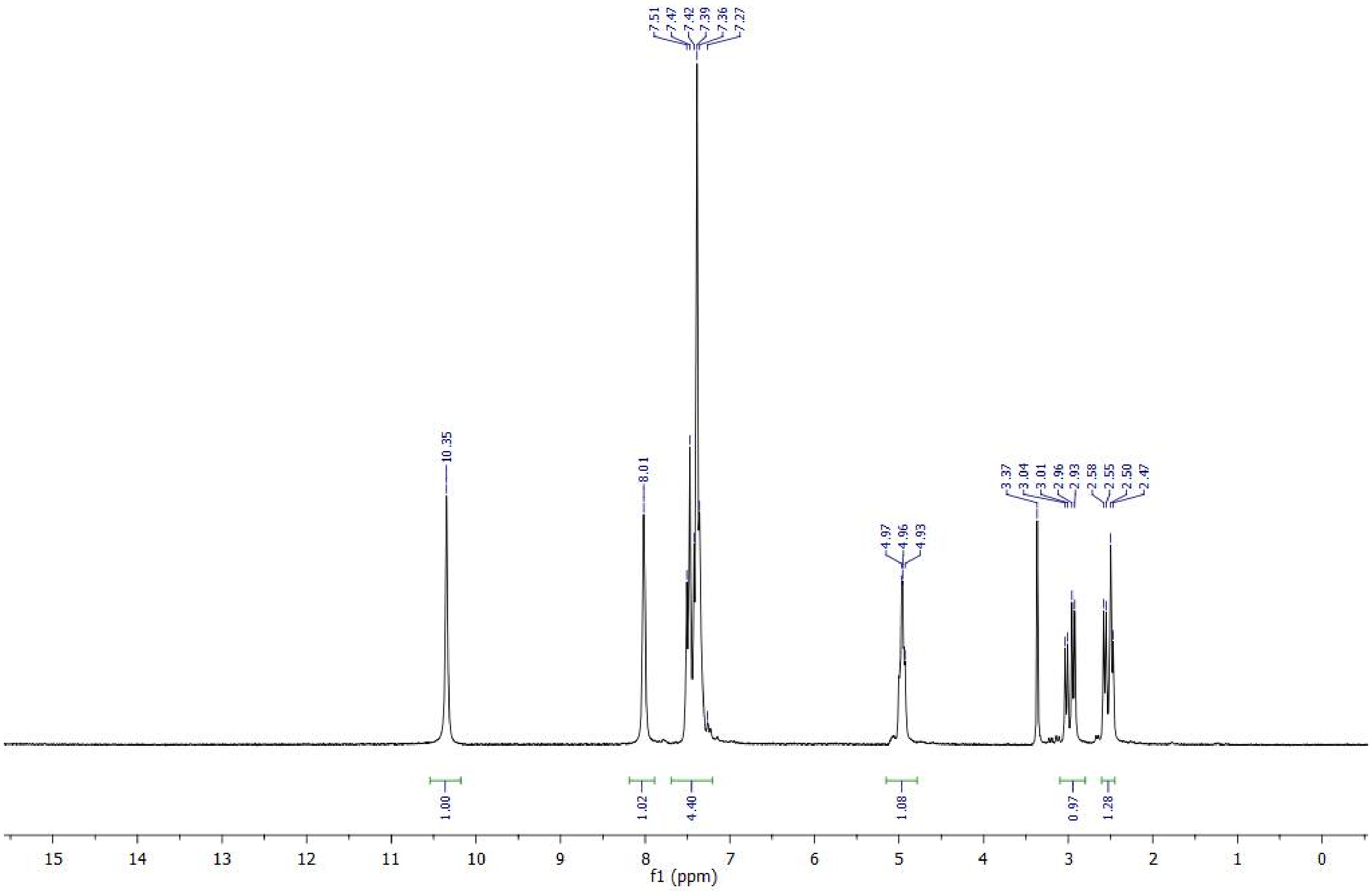

Figure S9. ^{1}H NMR spectrum of **2e**

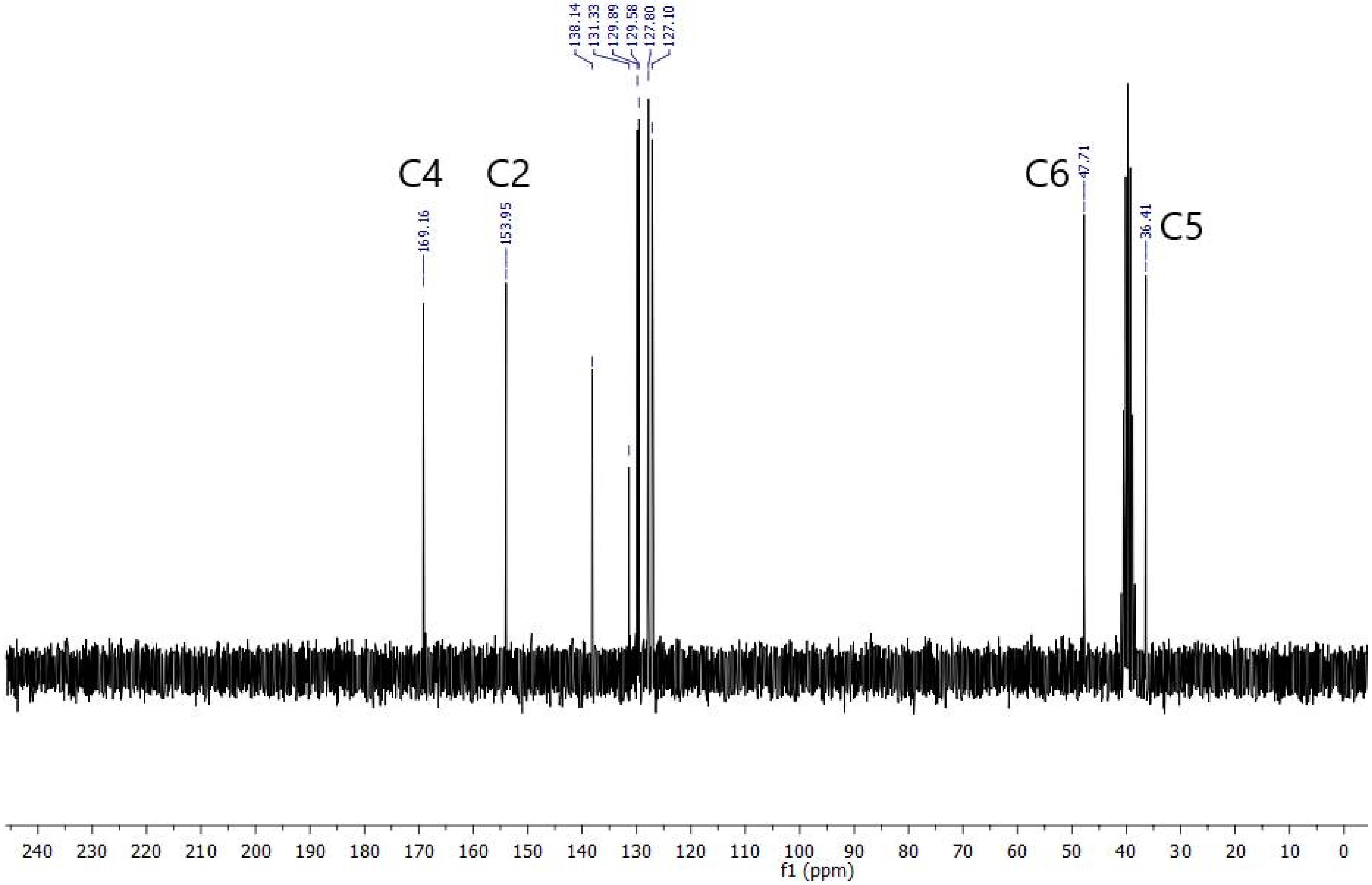

Figure S10. ^{13}C NMR spectrum of **2e**

6-(4’-chlorophenyl)-dihydropyrimidine-2,4(1*H*,3*H*)-dione **2f**

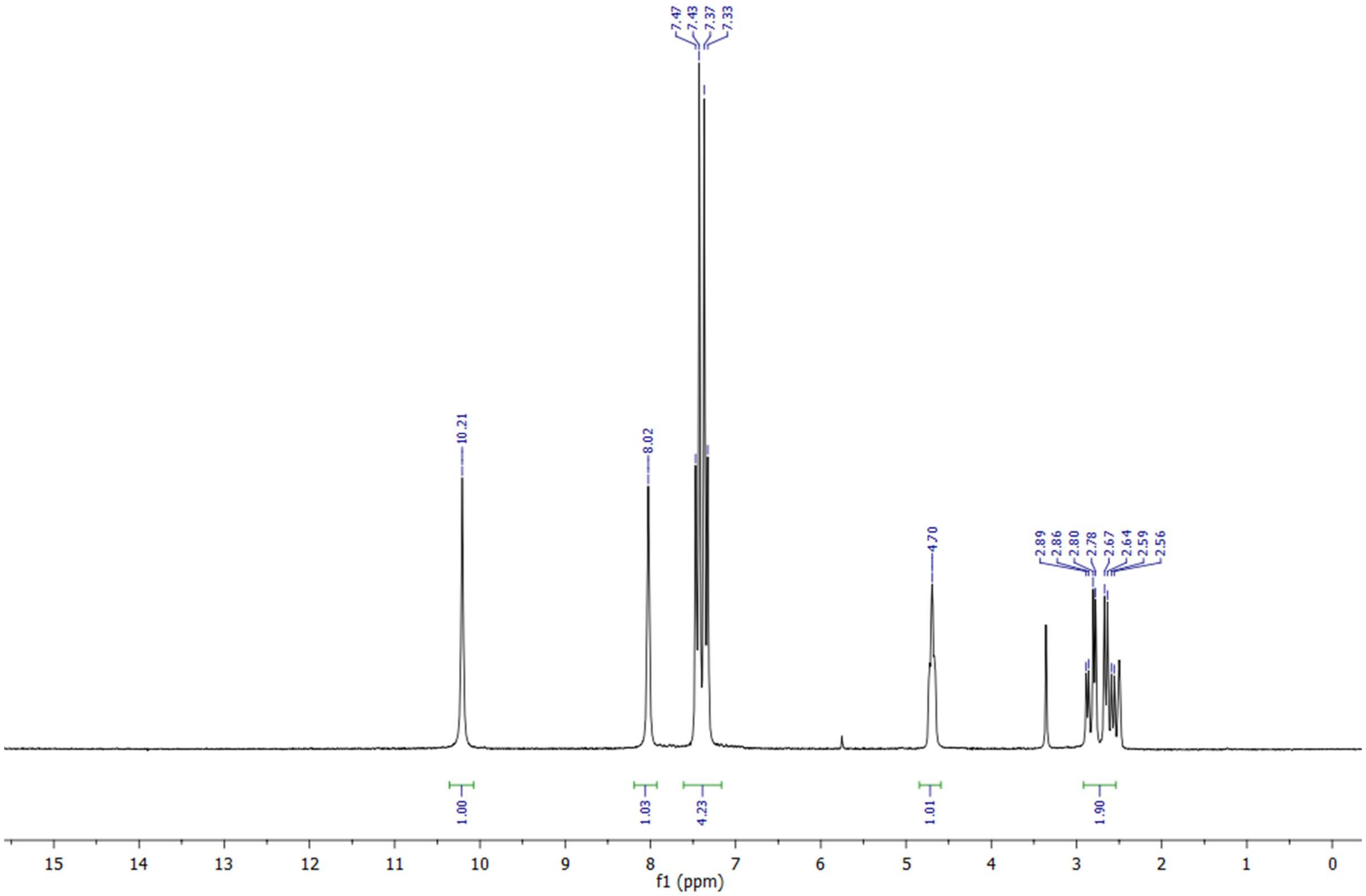

Figure S11. ^{1}H NMR spectrum of **2f**

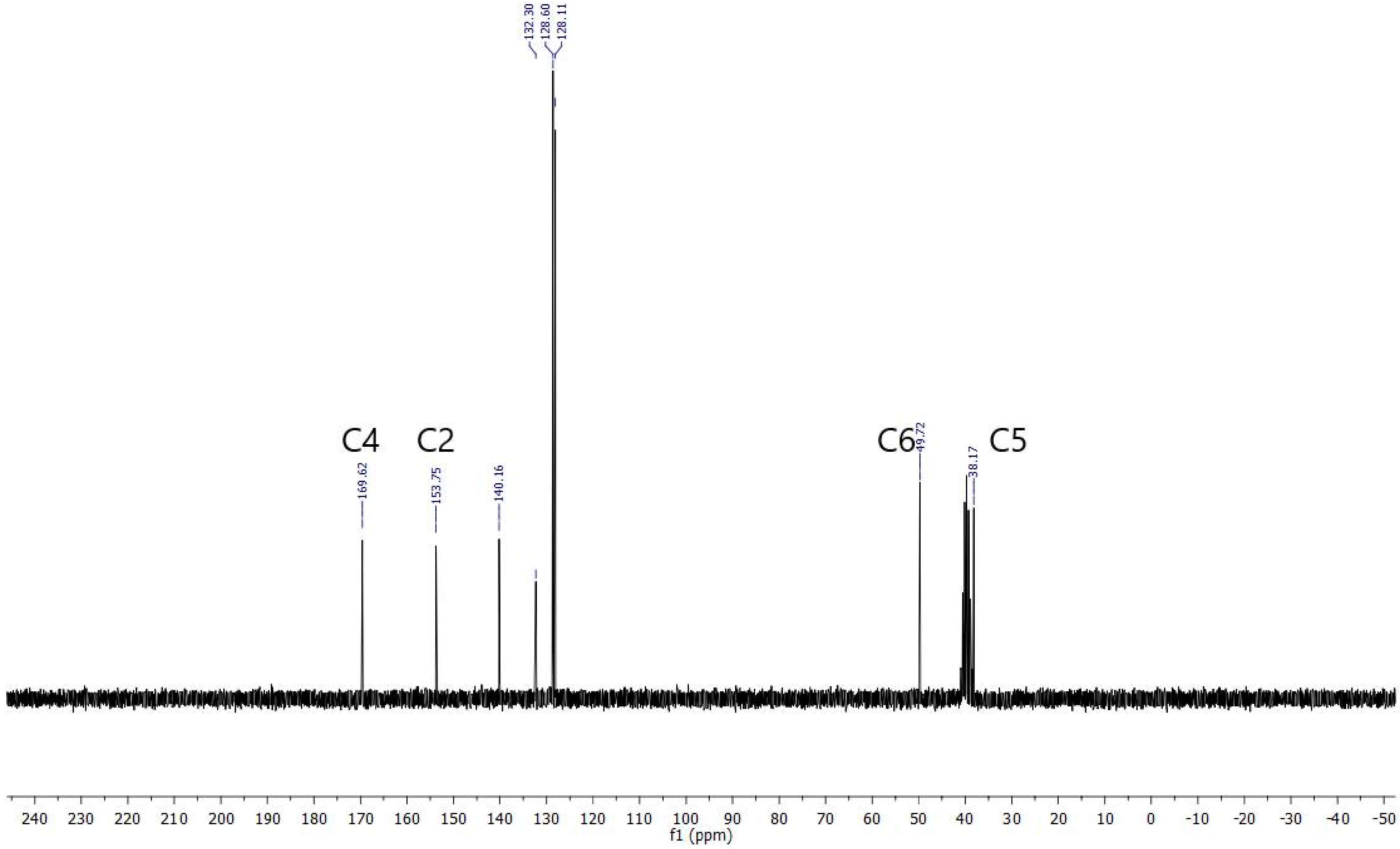

Figure S12. ^{13}C NMR spectrum of **2f**

6-(4’-nitrophenyl)-dihydropyrimidine-2,4(1*H*,3*H*)-dione **2g**

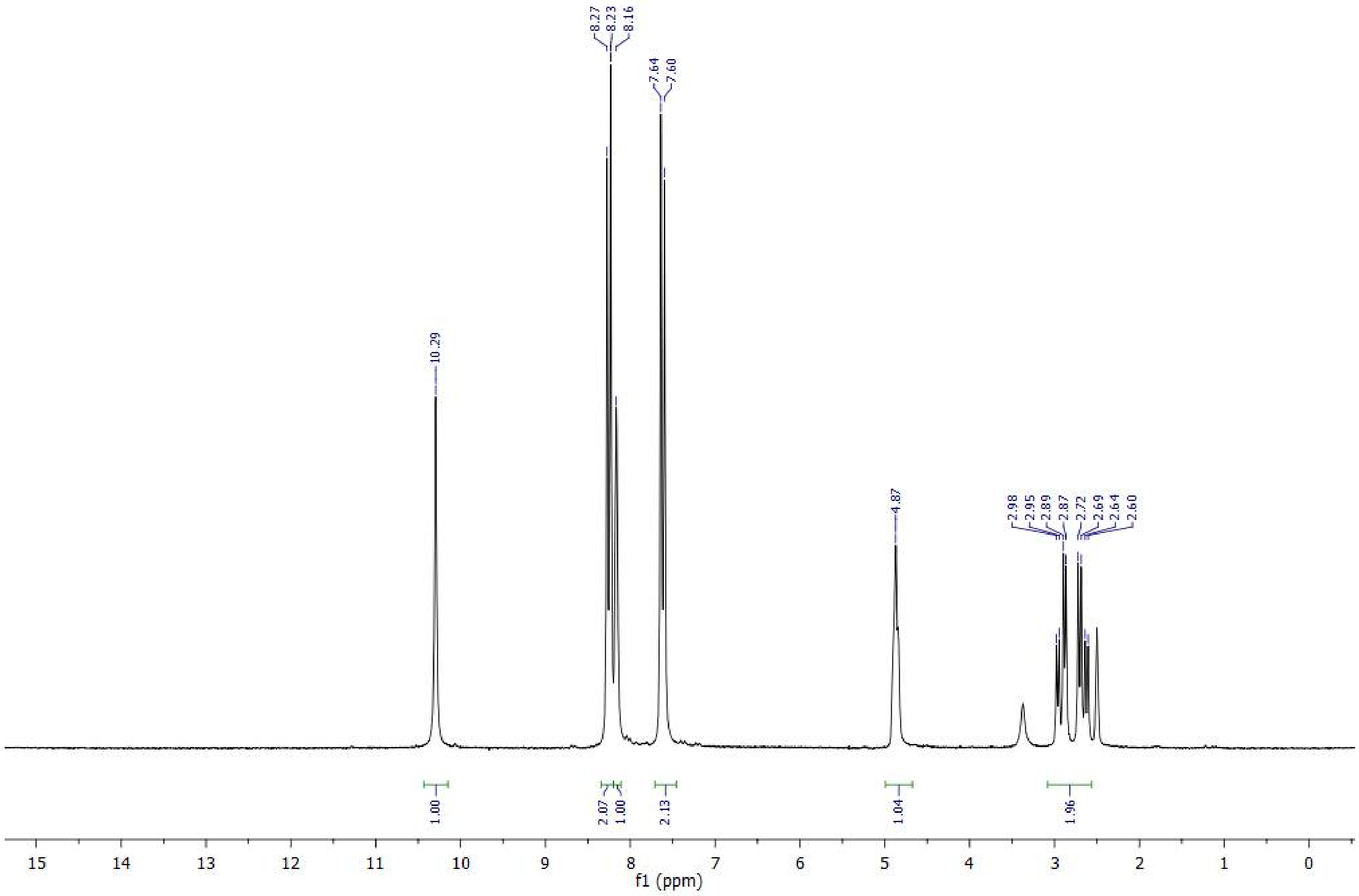

Figure S13. ^{1}H NMR spectrum of **2g**

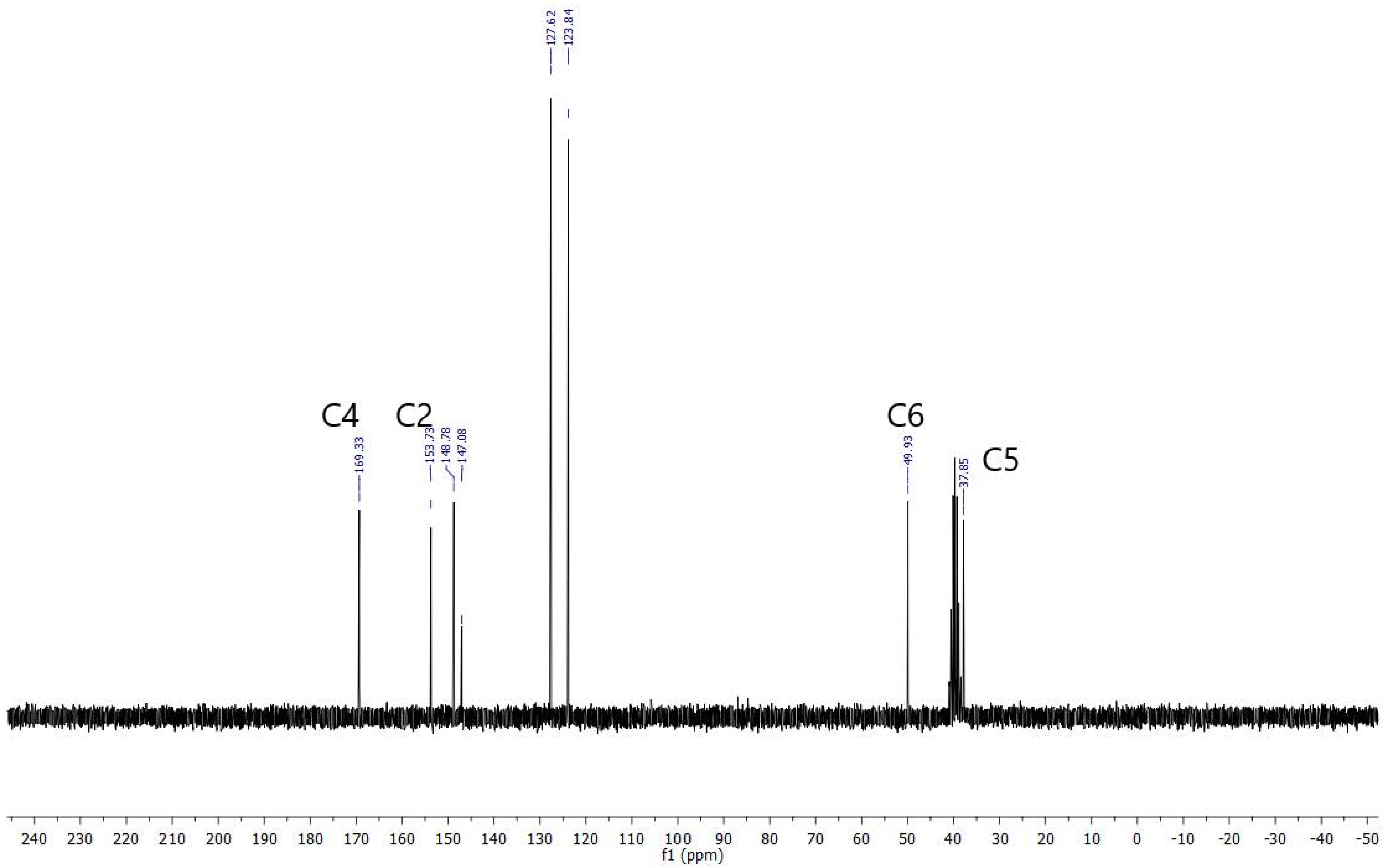

Figure S14. ^{13}C NMR spectrum of **2g**

6-(4'-benzyloxyphenyl)-dihydropyrimidine-2,4(1*H*,3*H*)-dione **2h**

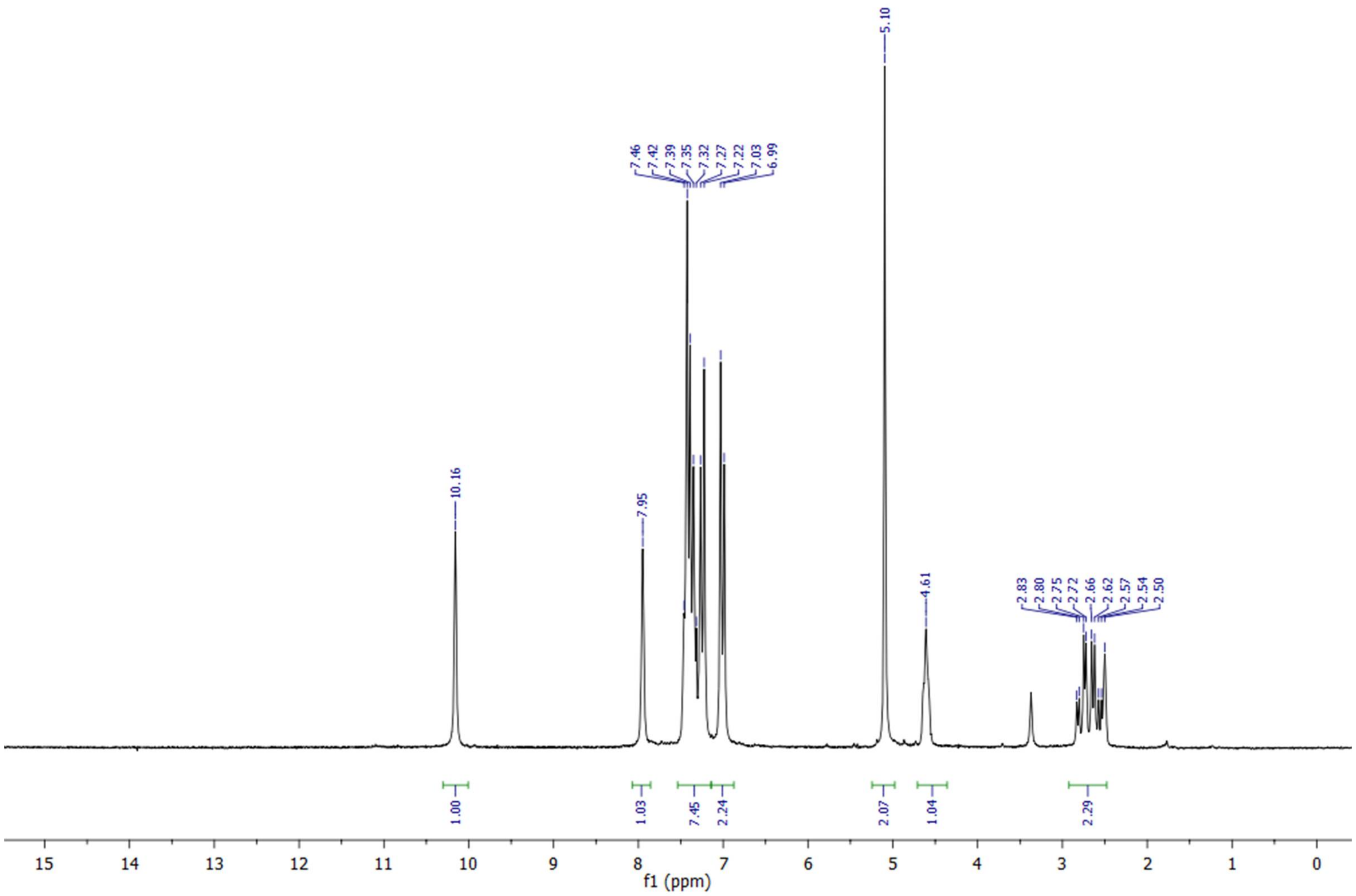

Figure S15. ^{1}H NMR spectrum of **2h**

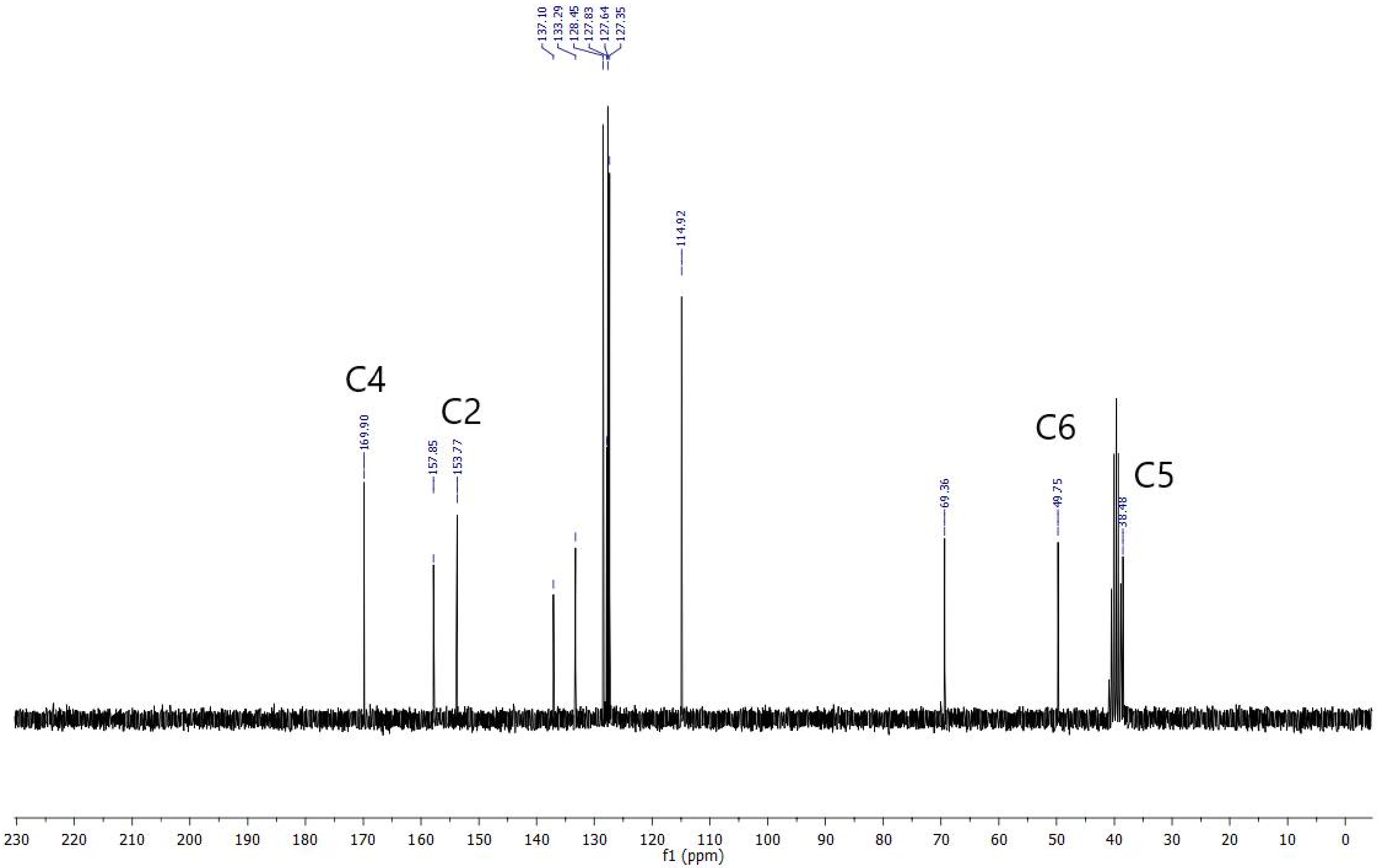

Figure S16. ^{13}C NMR spectrum of **2h**

6-(4’-(4’’-bromobenzyloxy)phenyl)-dihydropyrimidine-2,4(1*H*,3*H*)-dione **2i**

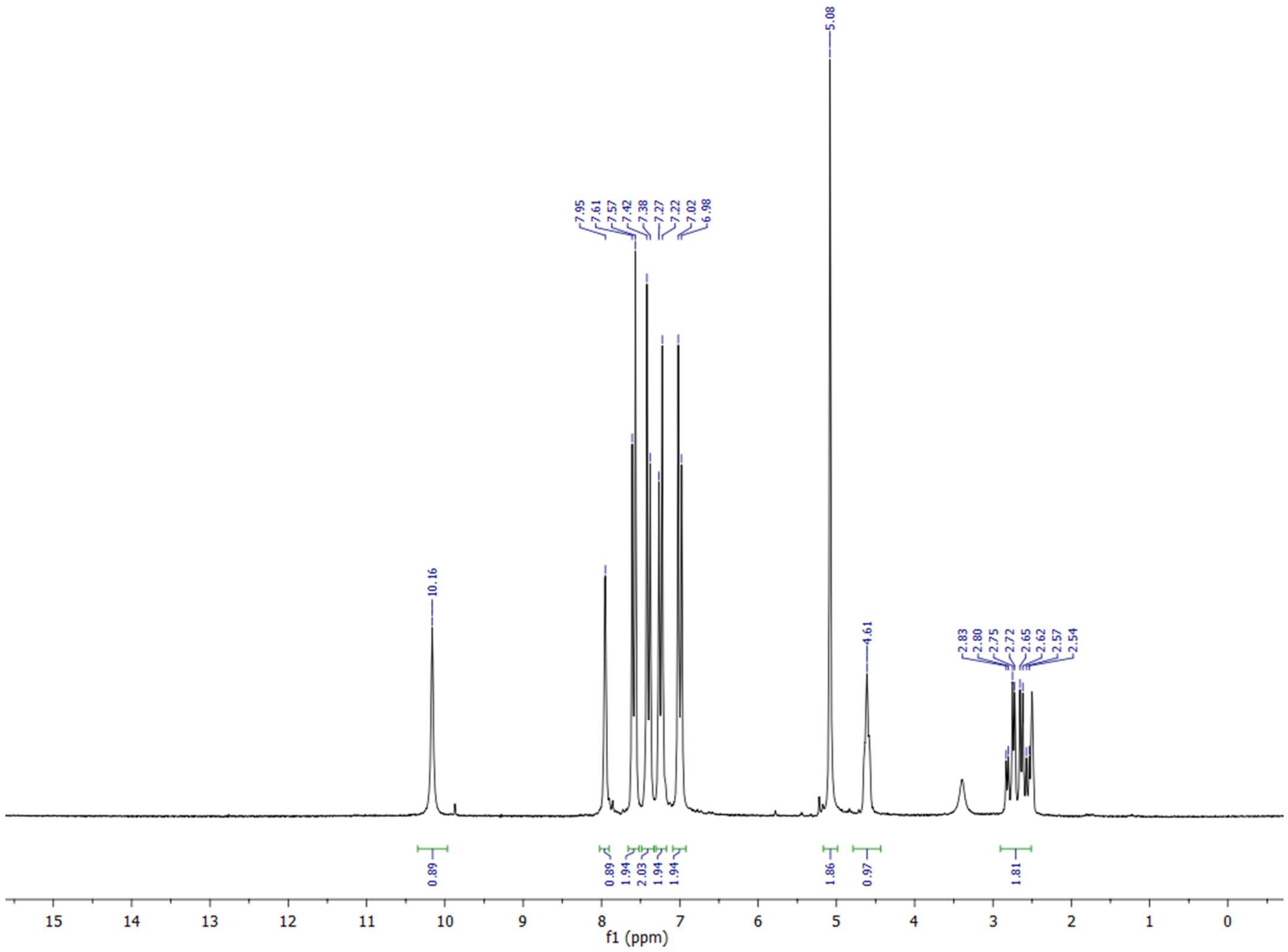

Figure S17. ^{1}H NMR spectrum of **2i**

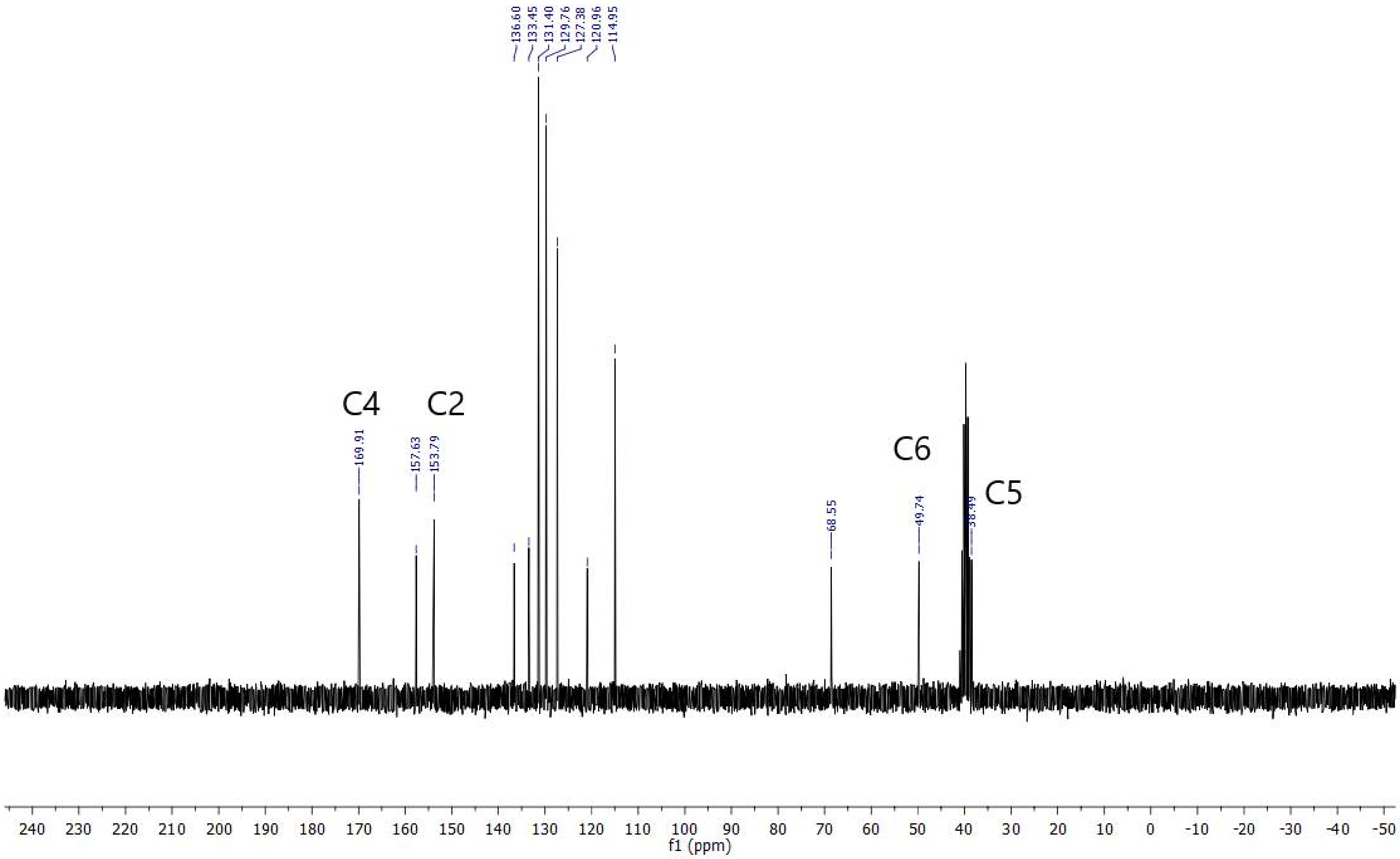

Figure S18. ^{13}C NMR spectrum of **2i**

dihydro-6-(4’-methoxyphenyl)pyrimidine-2,4(1H,3H)-dione **2j**

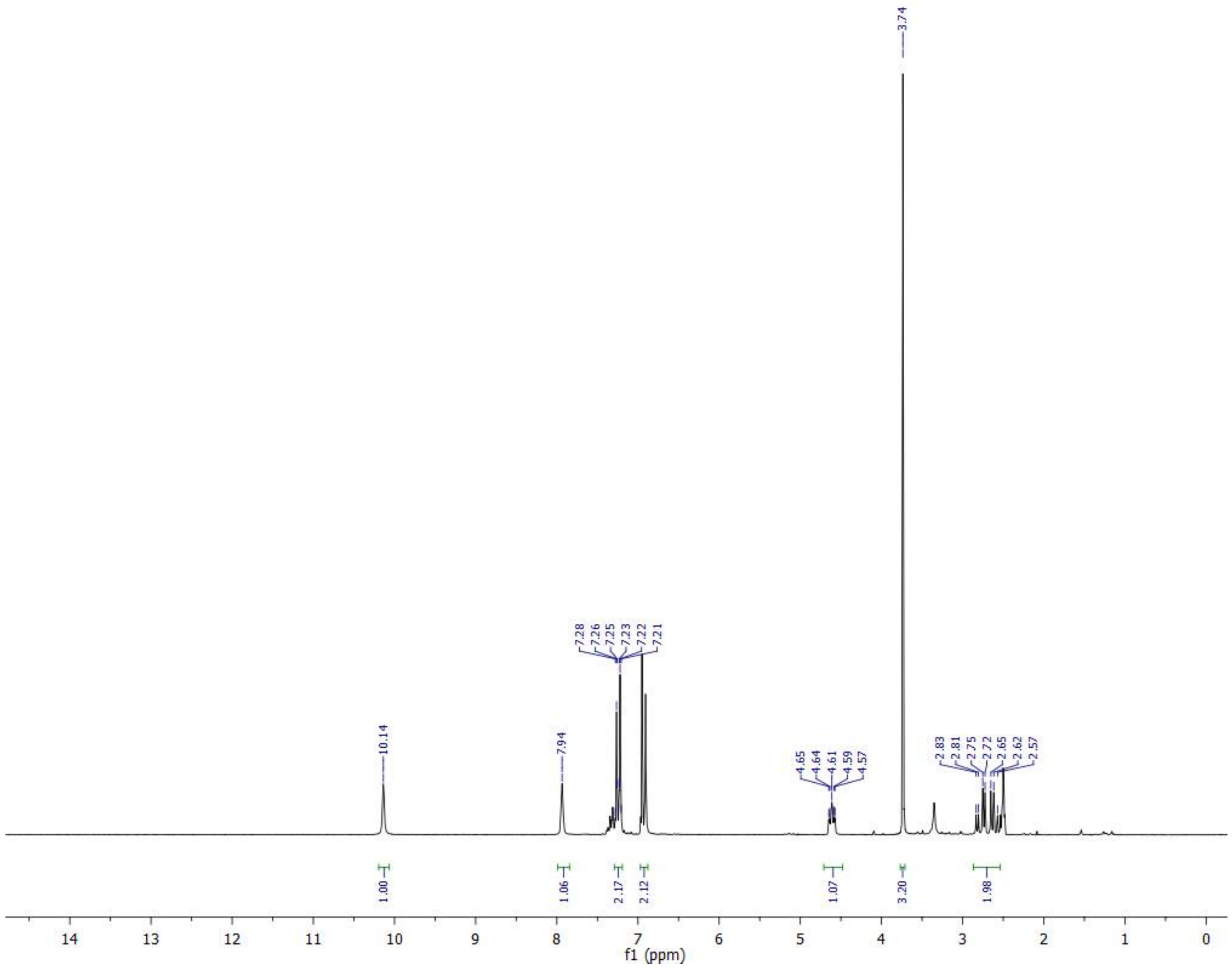

Figure S19. ^{1}H NMR spectrum of **2j**

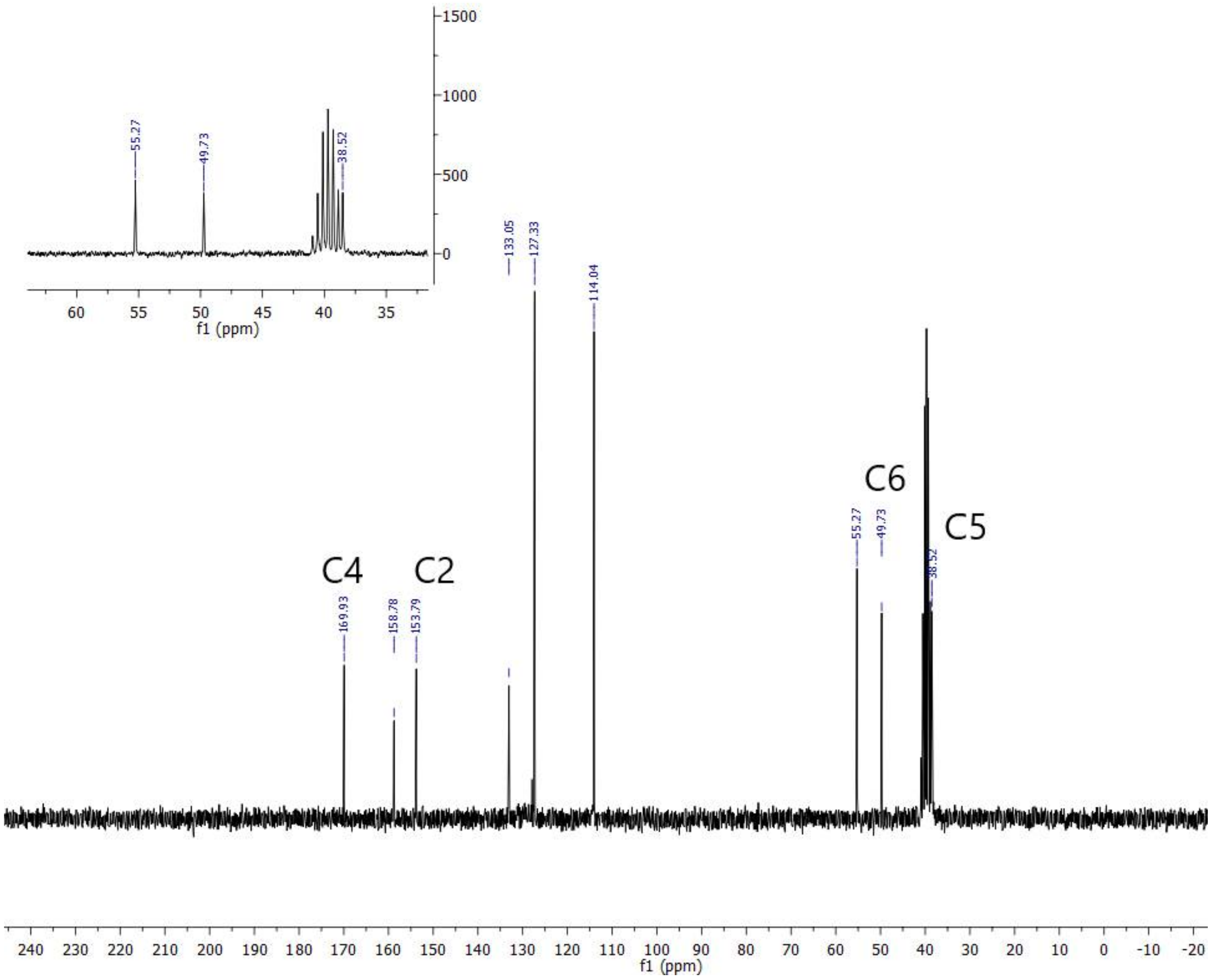

Figure S20. ^{13}C NMR spectrum of **2j**

6-(3’,4’-dimethoxyphenyl)-dihydropyrimidine-2,4(1*H*,3*H*)-dione **2k**

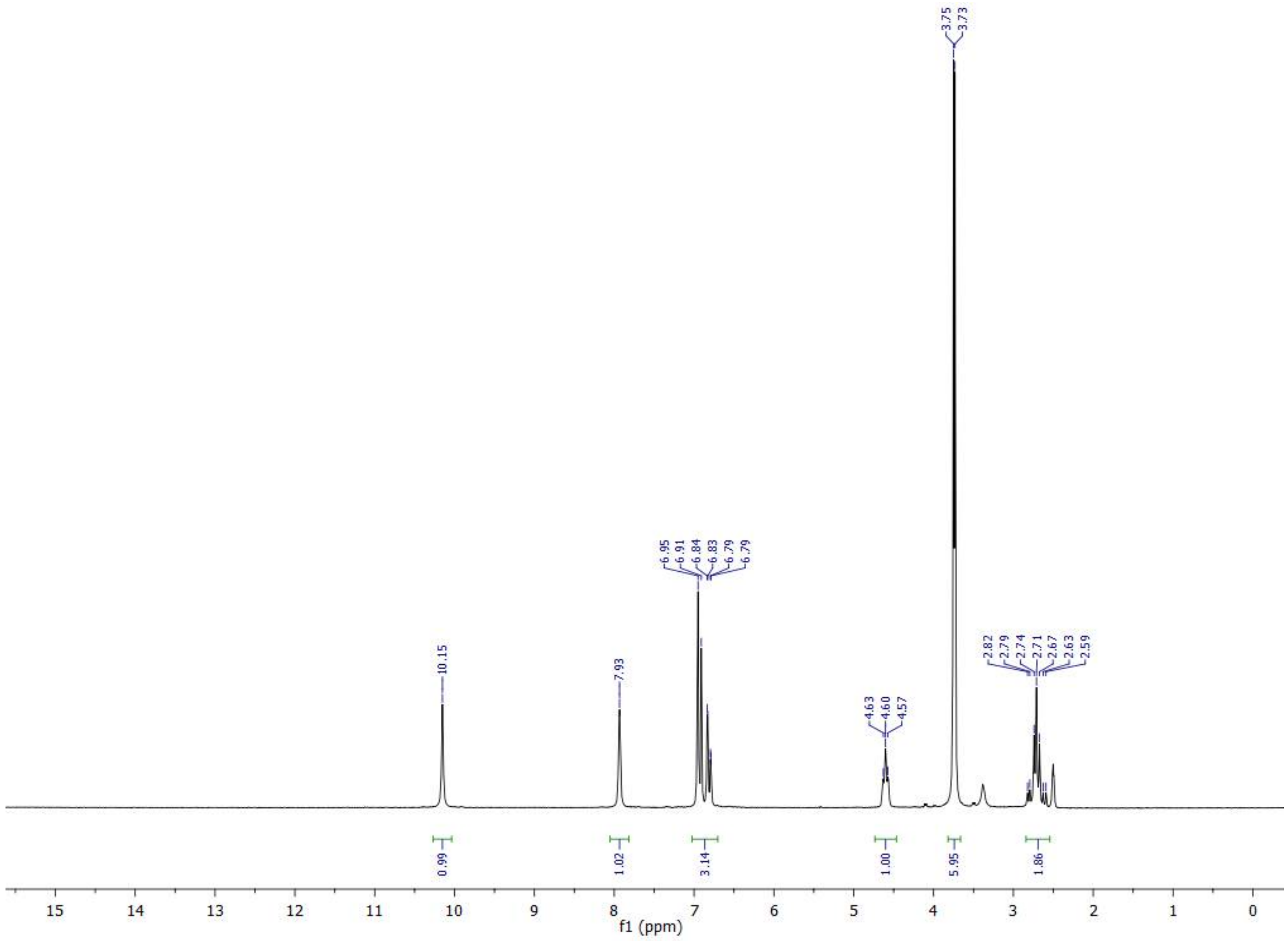

Figure S21. ^{1}H NMR spectrum of **2k**

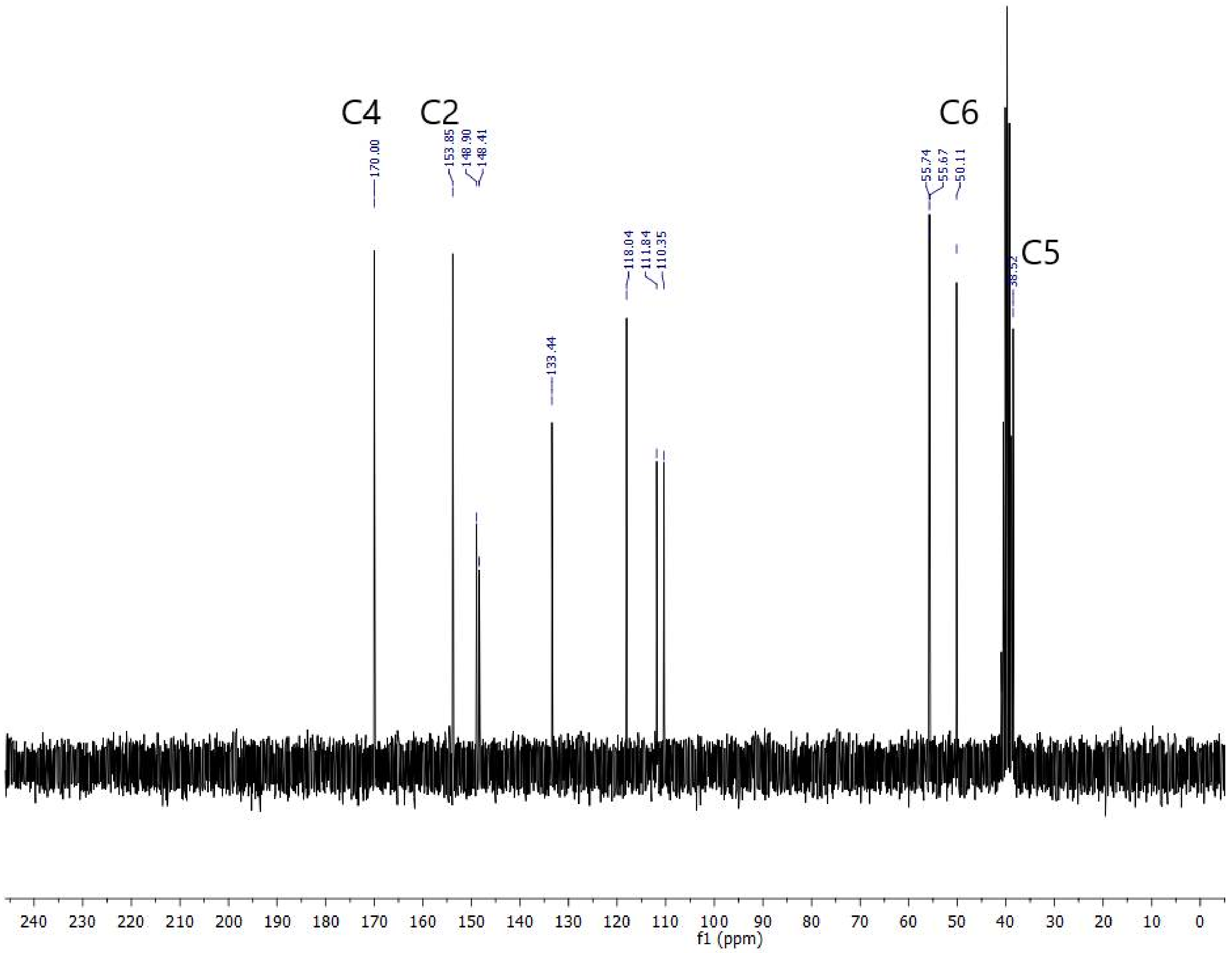

Figure S22. ^{13}C NMR spectrum of **2k**

dihydro-6-(3’,4’,5’-trimethoxyphenyl)pyrimidine-2,4(1H,3H)-dione **2l**

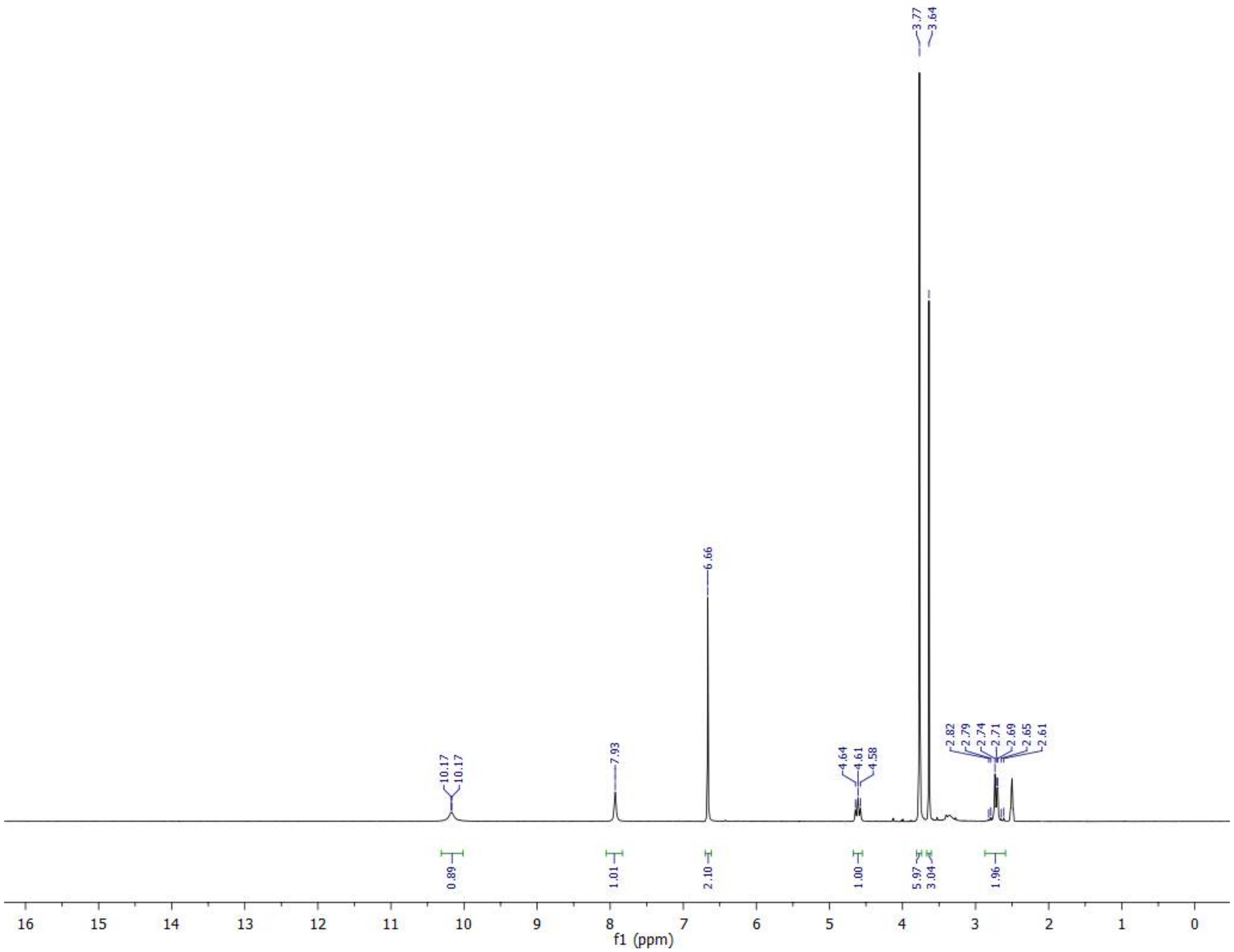

Figure S23. ^{1}H NMR spectrum of **2l**

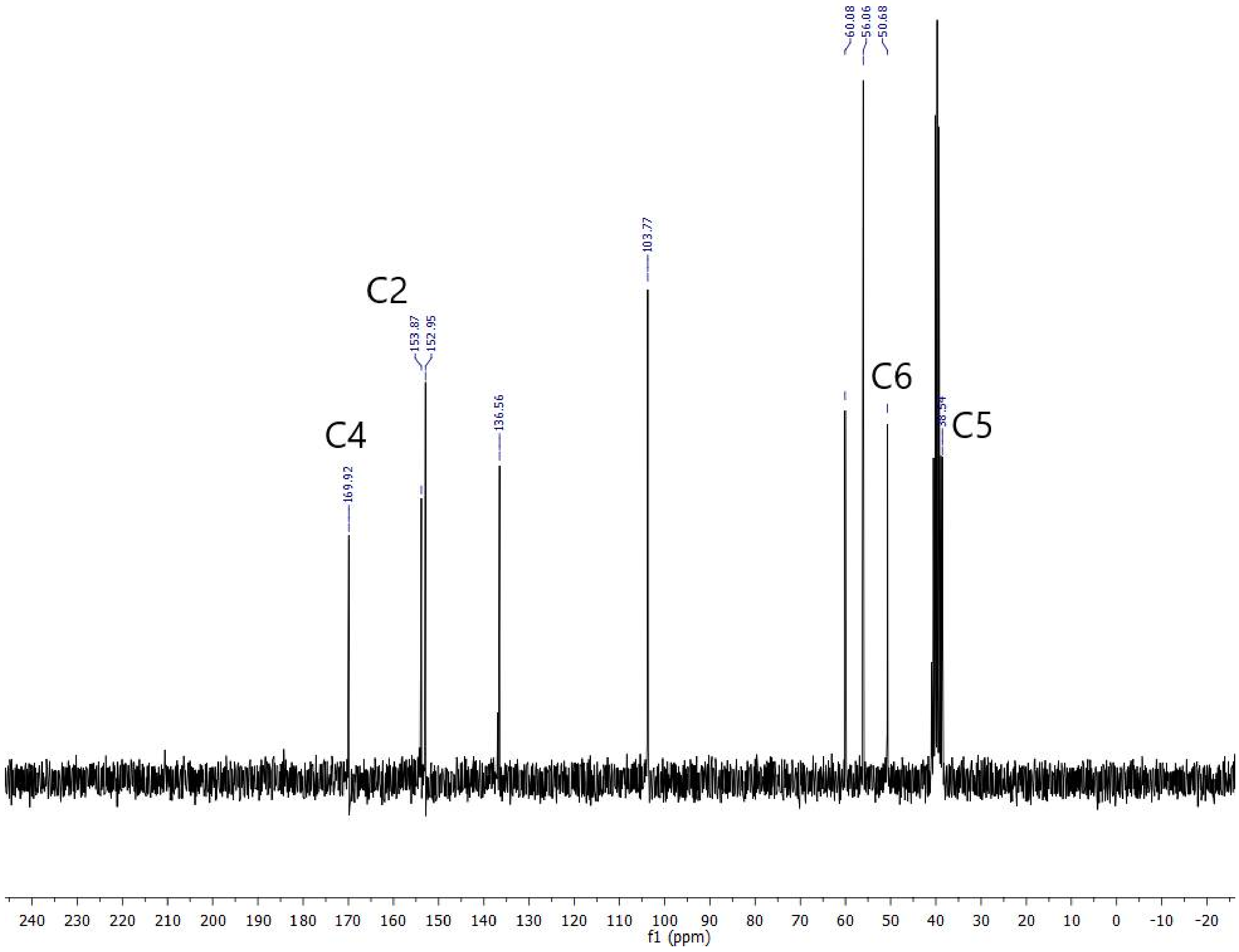

Figure S24. ^{13}C NMR spectrum of **2l**

6-(4’-ethoxy-3’-methoxyphenyl)-dihydropyrimidine-2,4(1*H*,3*H*)-dione **2m**

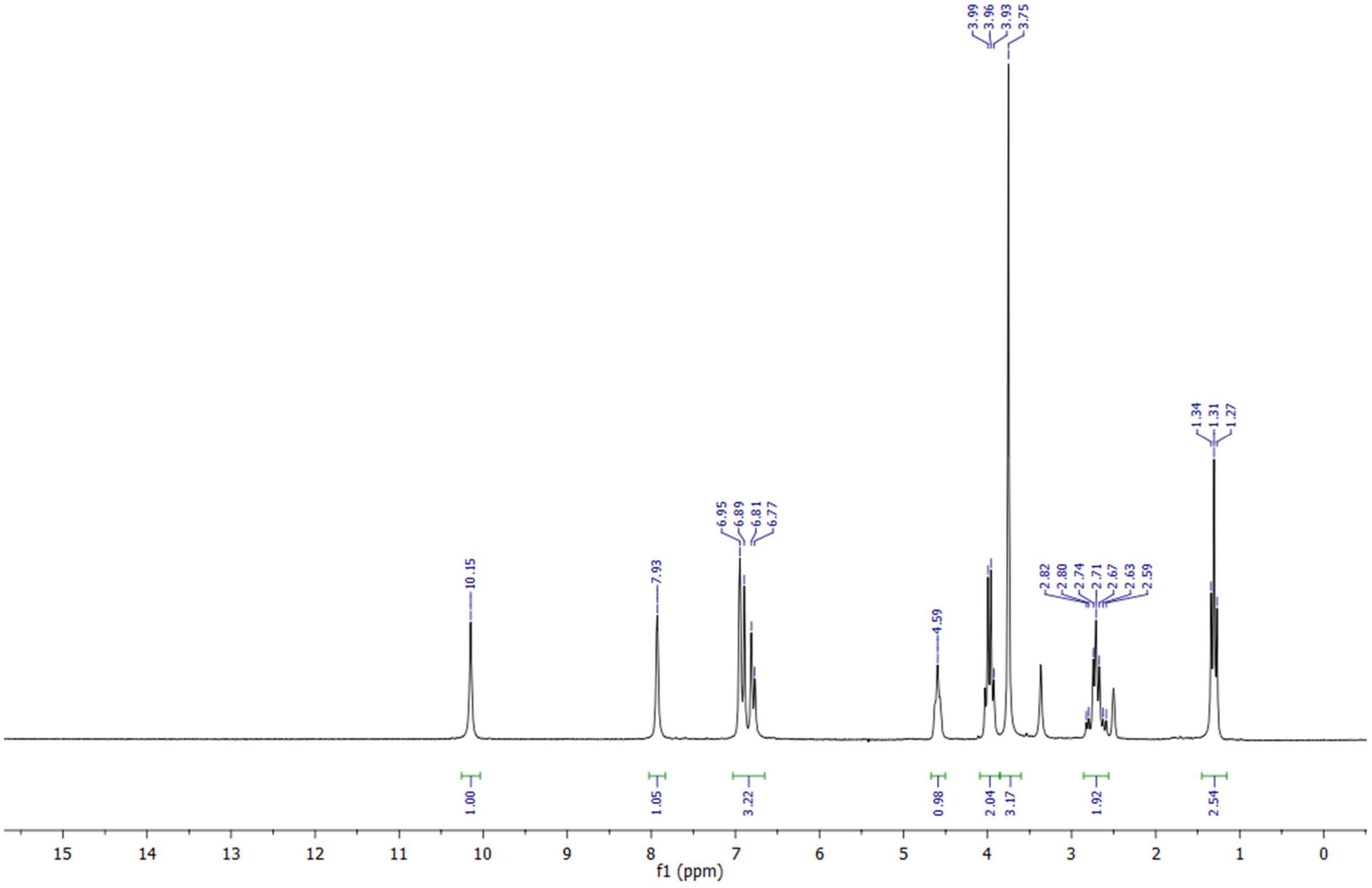

Figure S25. ^{1}H NMR spectrum of **2m**

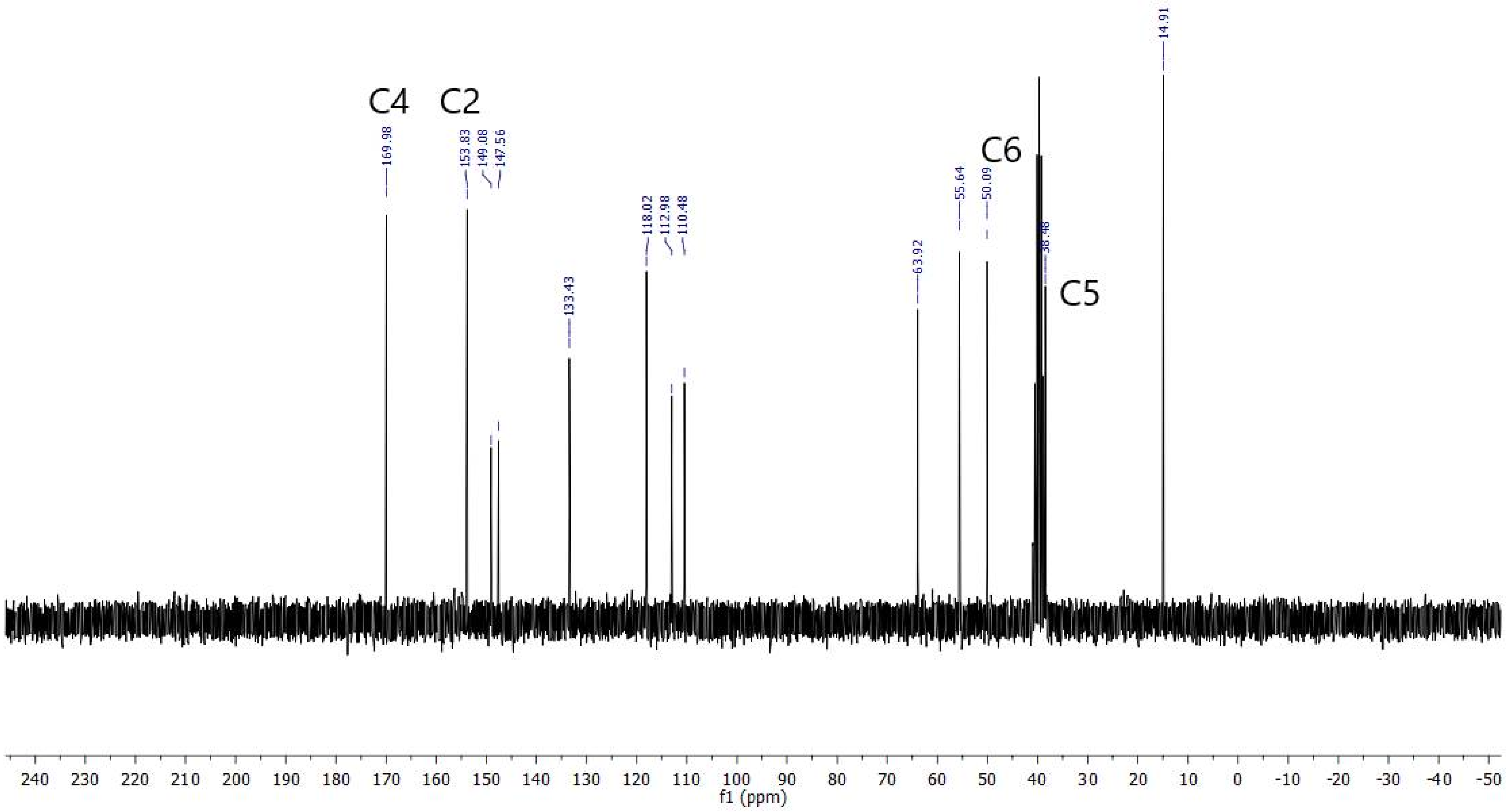

Figure S26. ^{13}C NMR spectrum of **2m**

6-(4’-propoxy-3’-methoxyphenyl)-dihydropyrimidine-2,4(1H,3H)-dione ***2n***

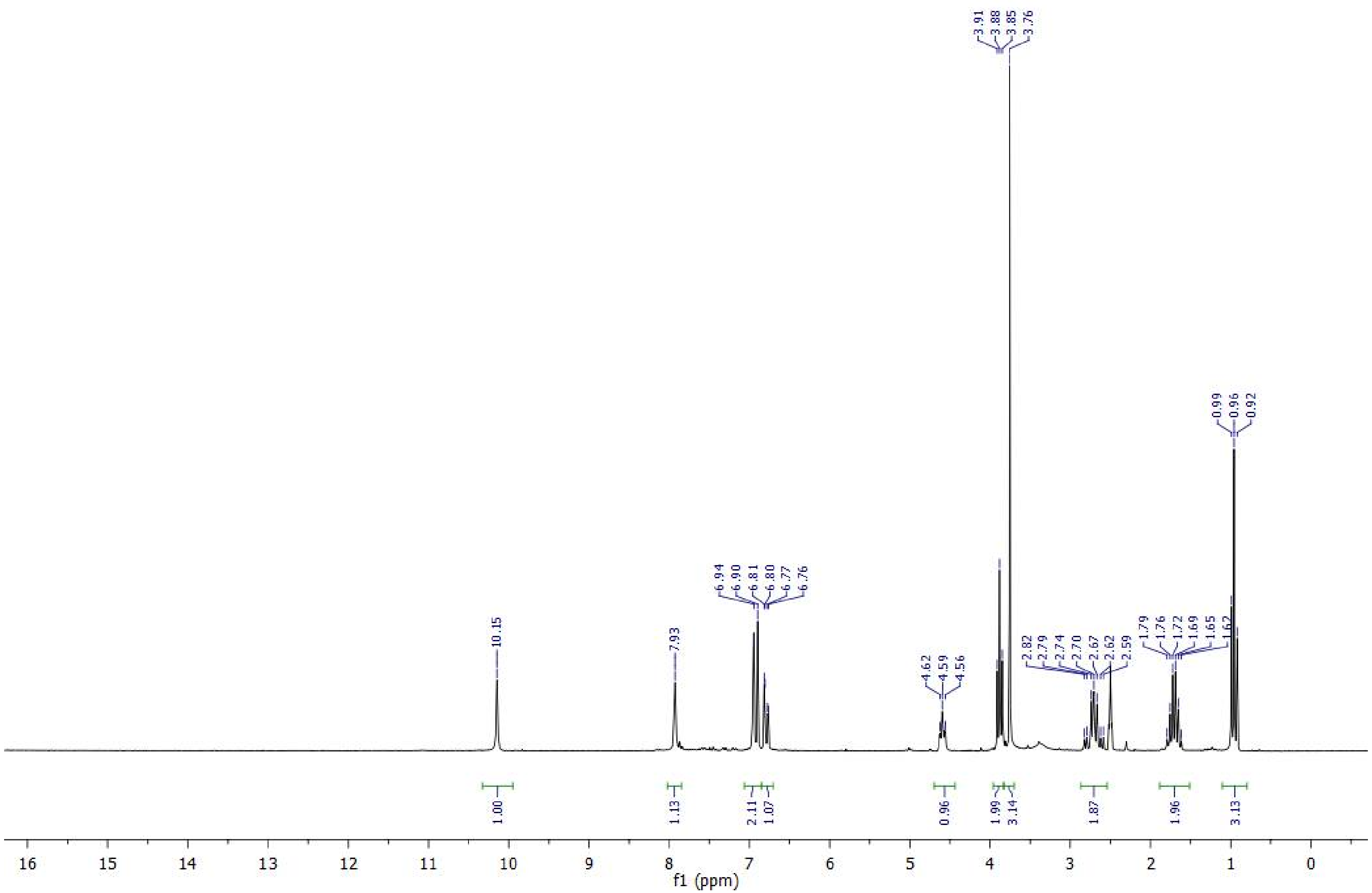

Figure S27. ^{1}H NMR spectrum of **2n**

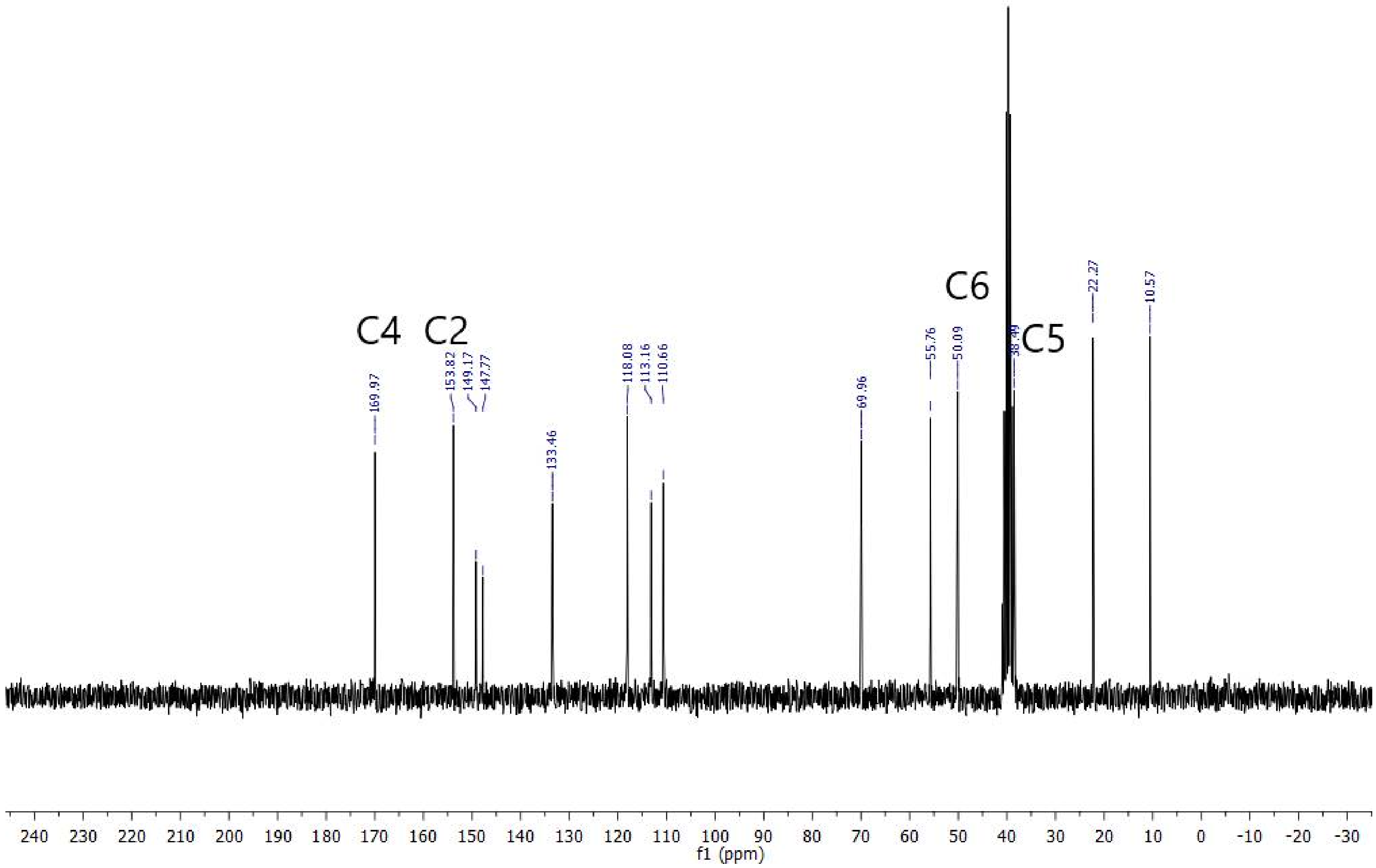

Figure S28. ^{13}C NMR spectrum of **2n**

6-(4’-butoxy-3’-methoxyphenyl)-dihydropyrimidine-2,4(1*H*,3*H*)-dione **2o**

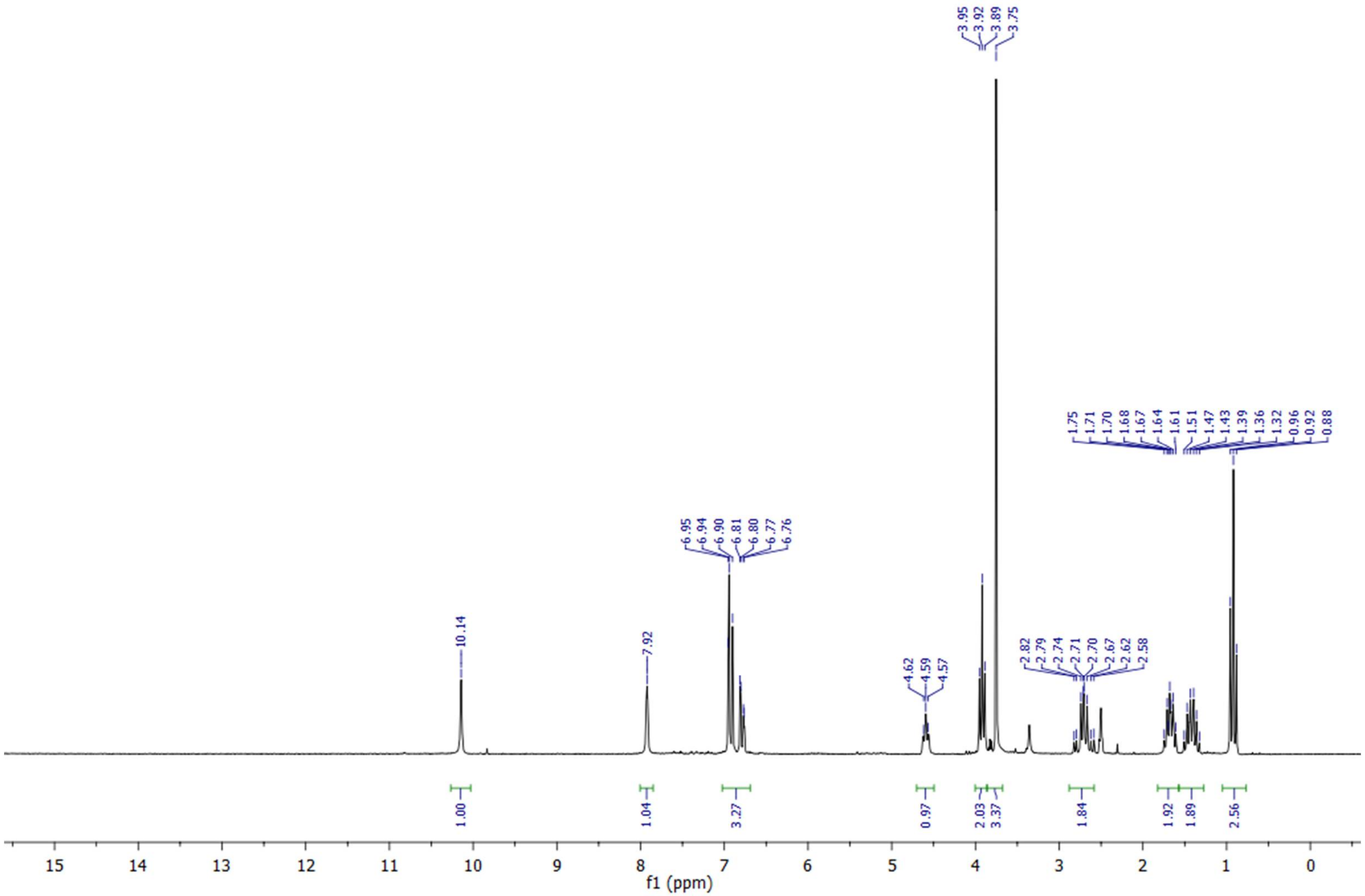

Figure S29. ^{1}H NMR spectrum of **2o**

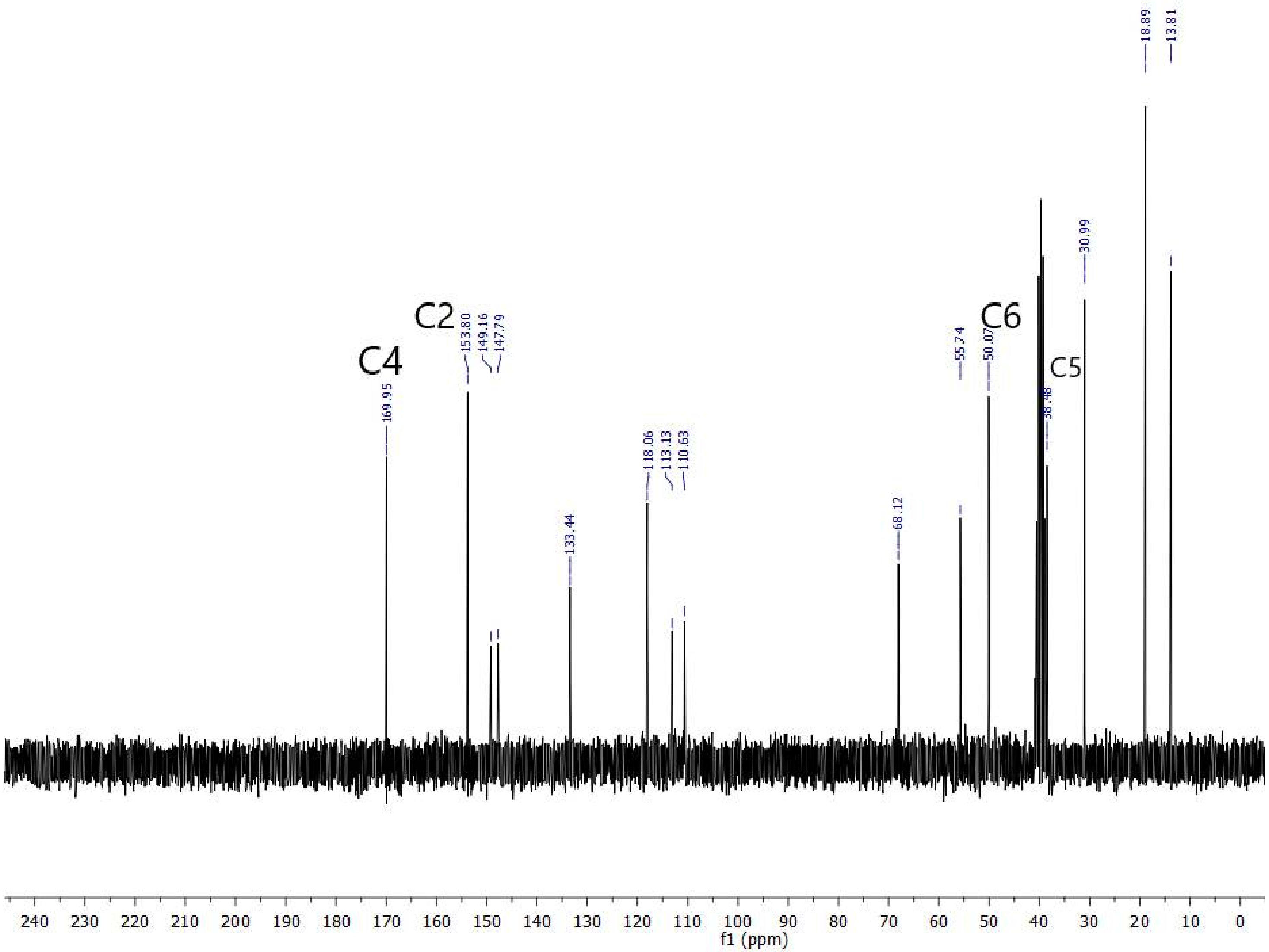

Figure S30. ^{13}C NMR spectrum of **2o**

6-(4'-benzyloxy-3'-methoxyphenyl)-dihydropyrimidine-2,4(1*H*,3*H*)-dione **2p**

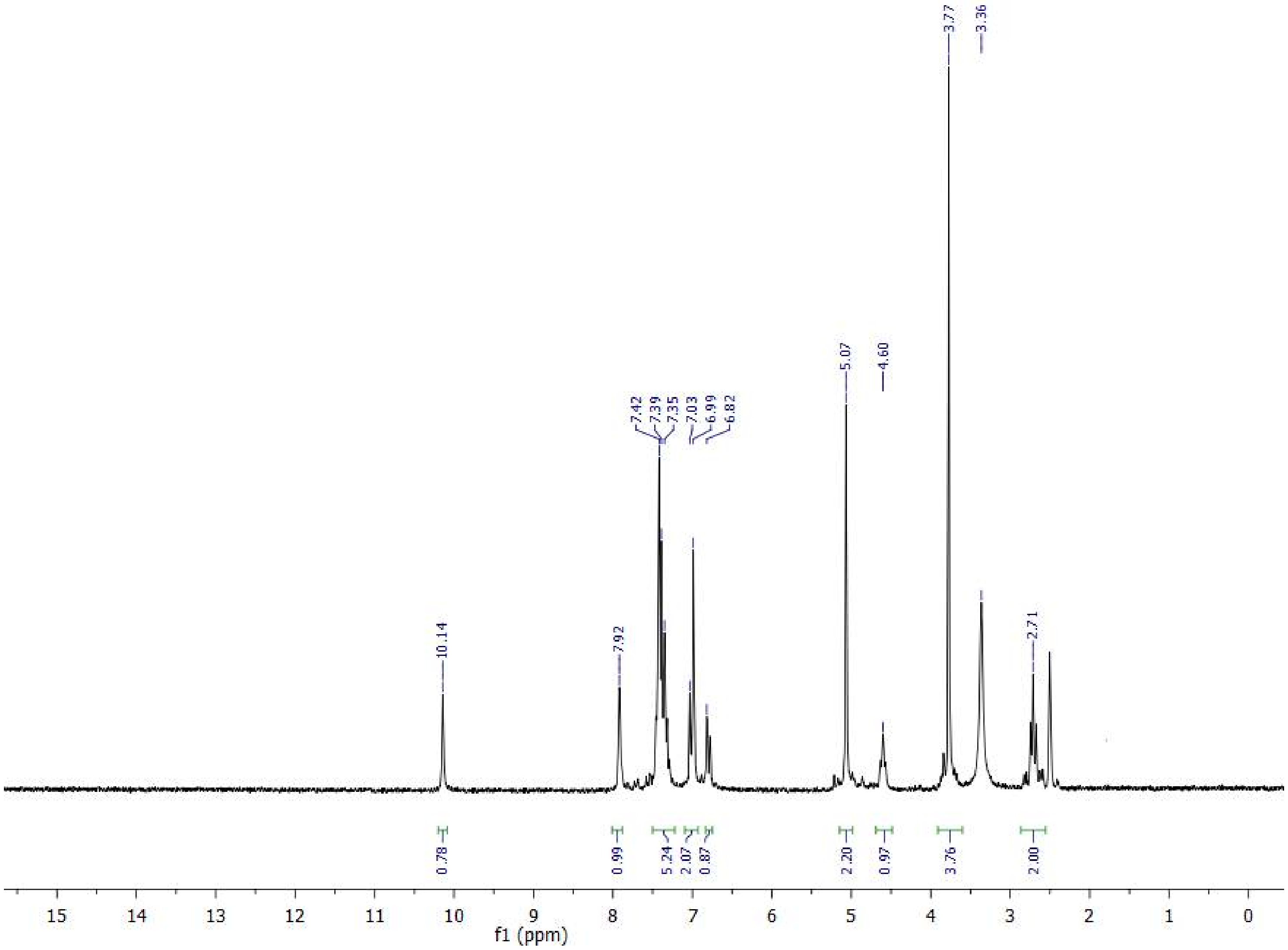

Figure S31. ^{1}H NMR spectrum of **2p**

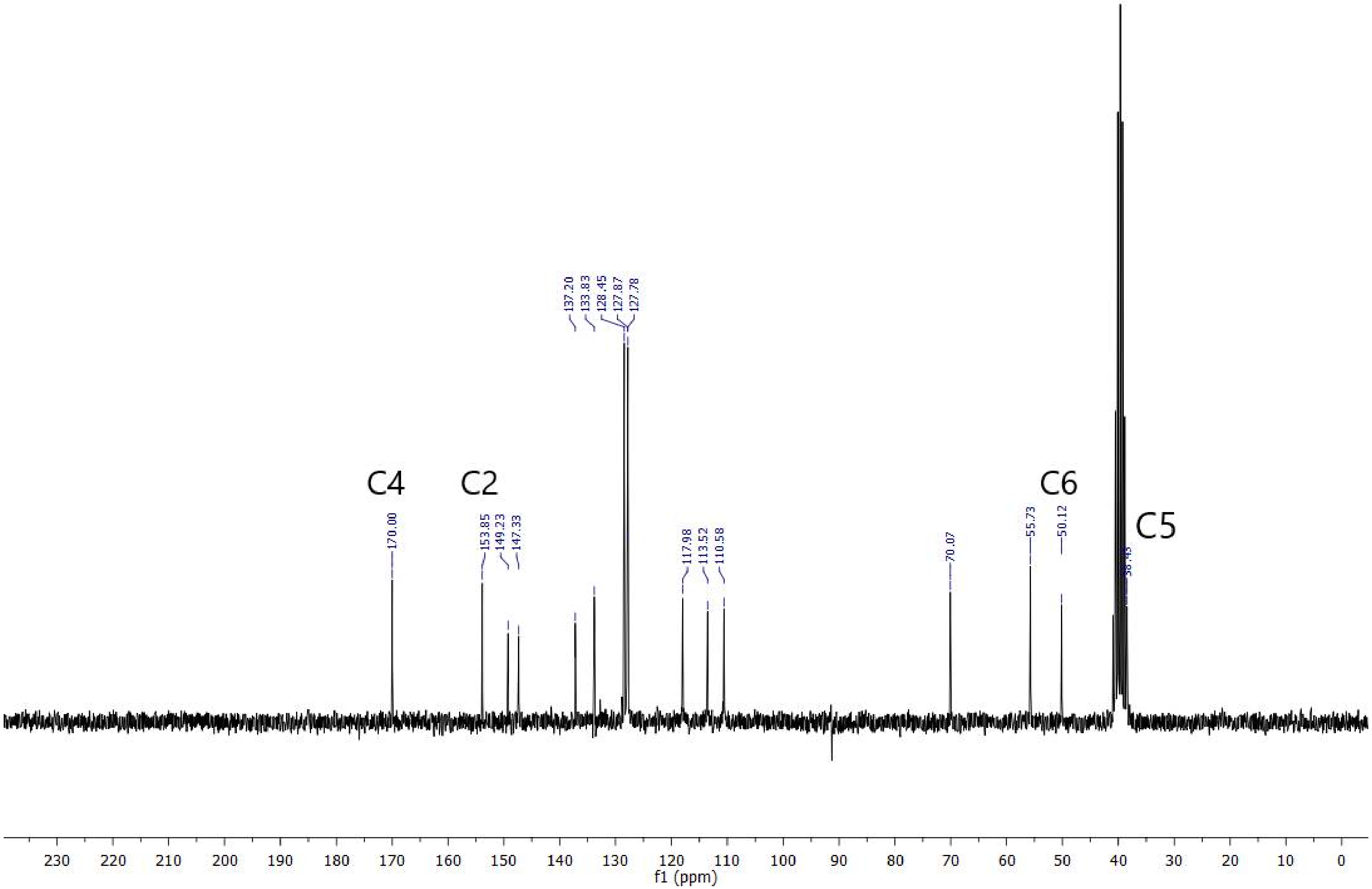

Figure S32. ^{13}C NMR spectrum of **2p**

6-(4'-(3''-methylbenzyloxy)-3'-methoxyphenyl)-dihydropyrimidine-2,4(1*H*,3*H*)-dione **2q**

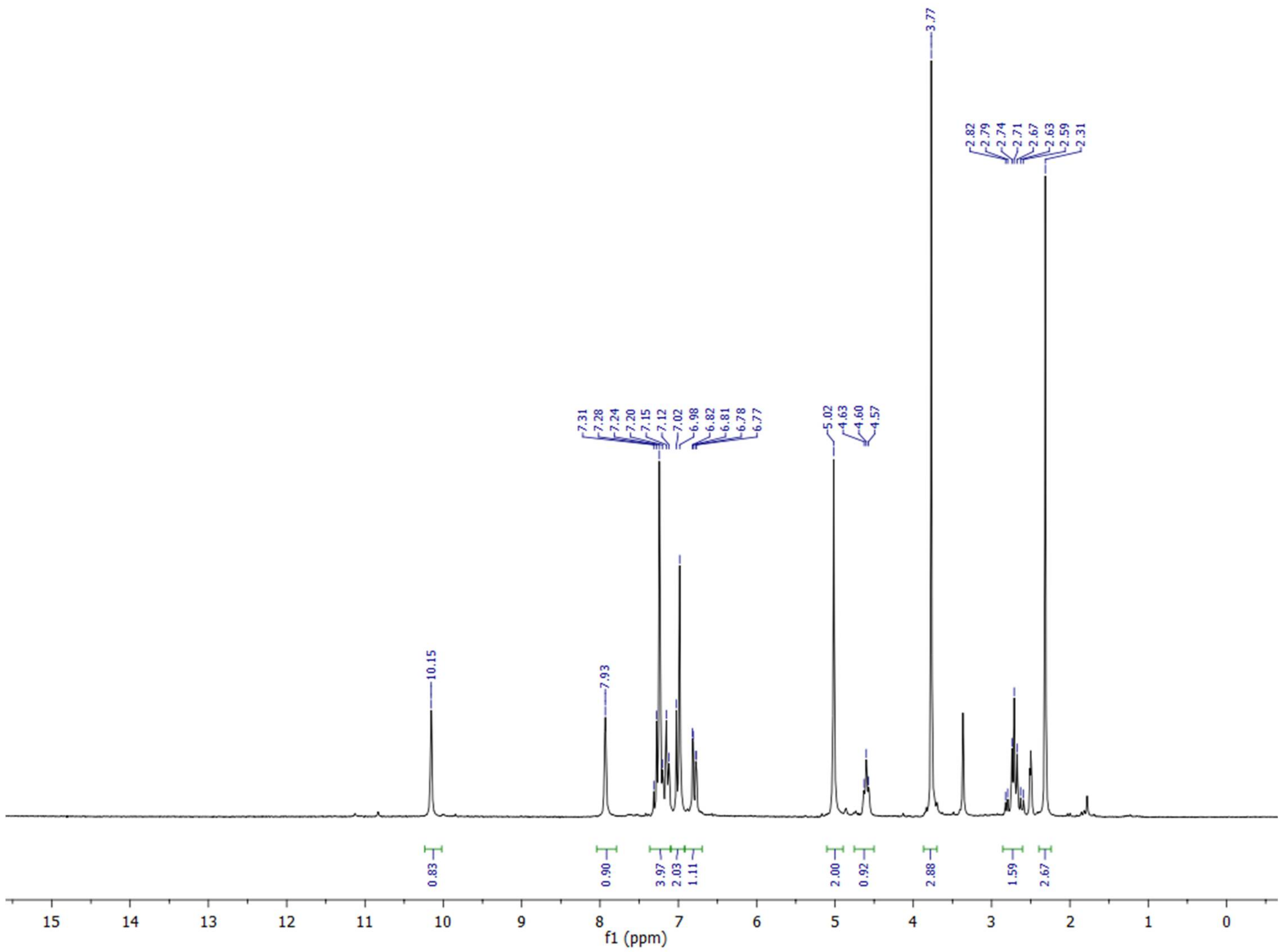

Figure S33. ^{1}H NMR spectrum of **2q**

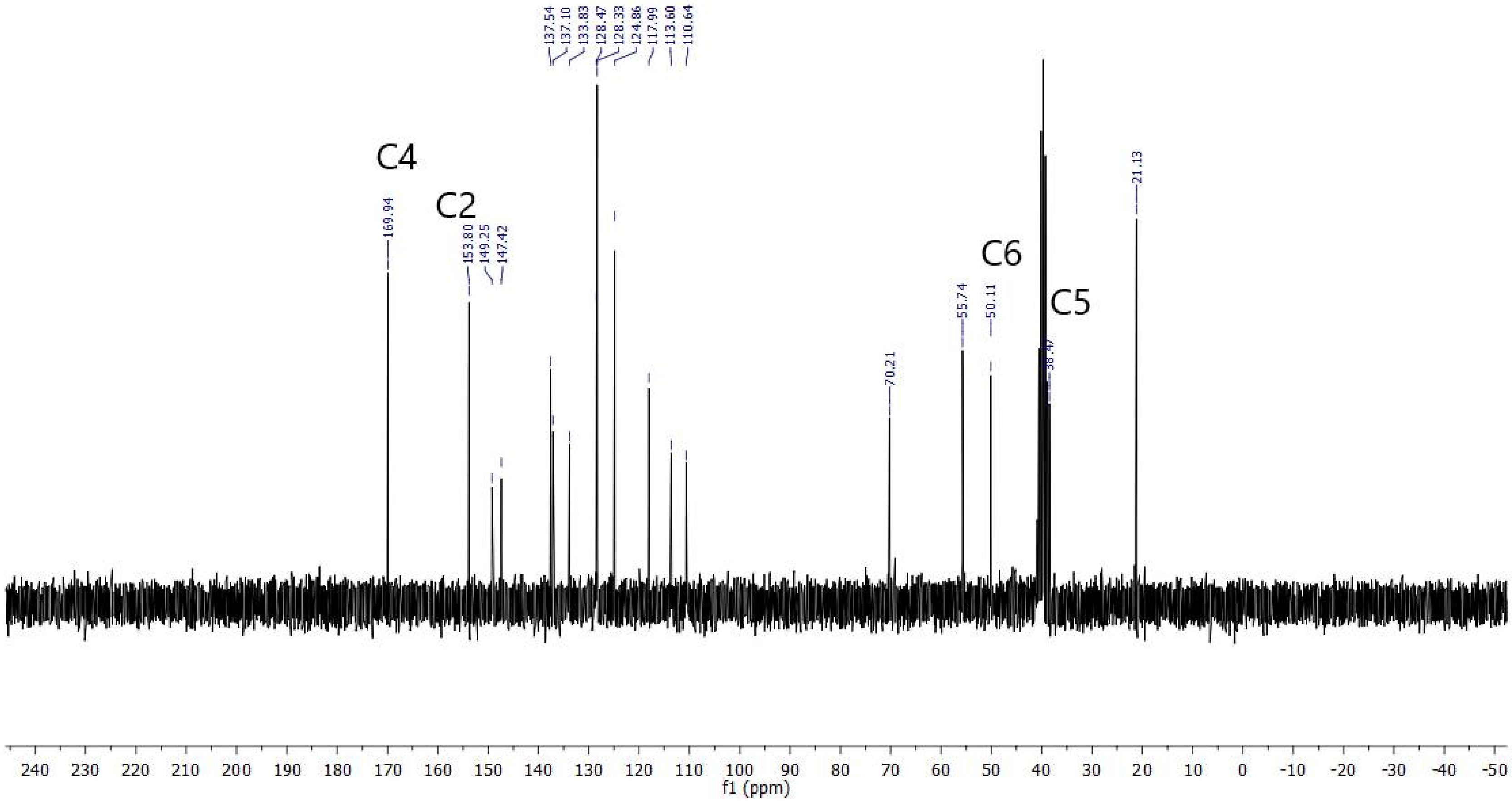

Figure S34. ^{13}C NMR spectrum of **2q**

6-(4'-(4''-methylbenzyloxy)-3'-methoxyphenyl)-dihydropyrimidine-2,4(1H,3H)-dione **2r**

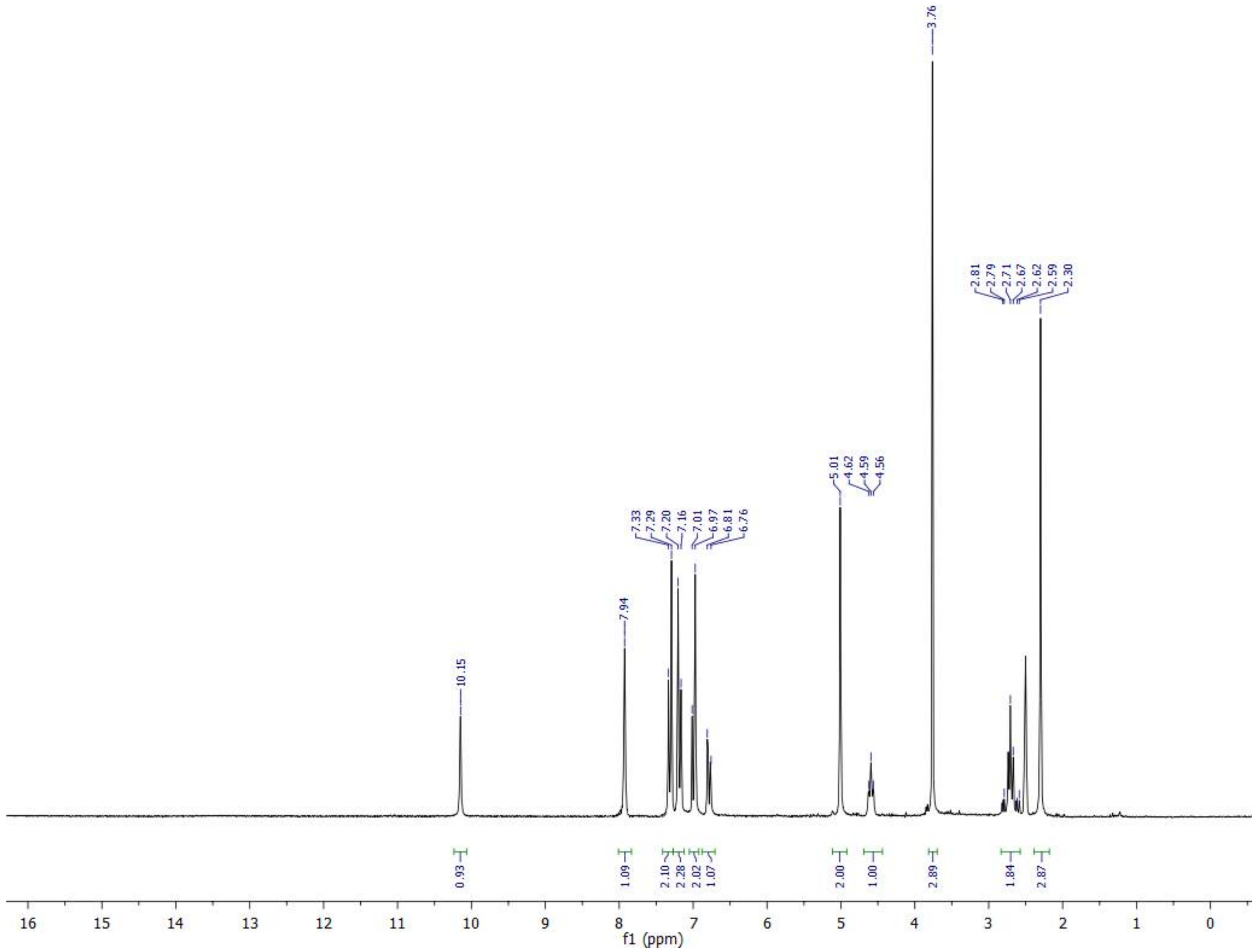

Figure S35. ^{1}H NMR spectrum of **2r**

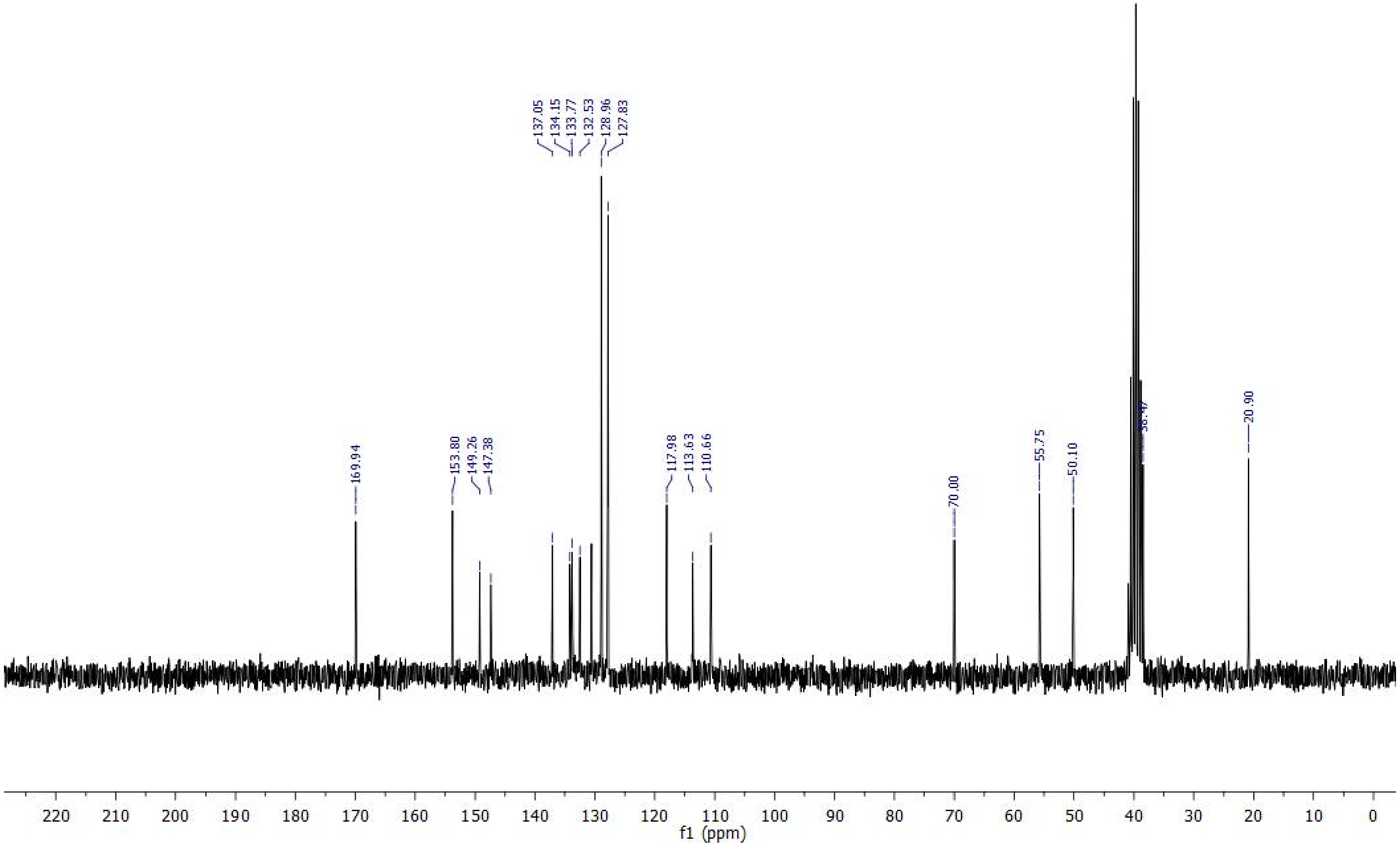

Figure S36. ^{13}C NMR spectrum of **2r**

6-(4'-acetoxy-3'-methoxyphenyl)-dihydropyrimidine-2,4(1*H*,3*H*)-dione **2s**

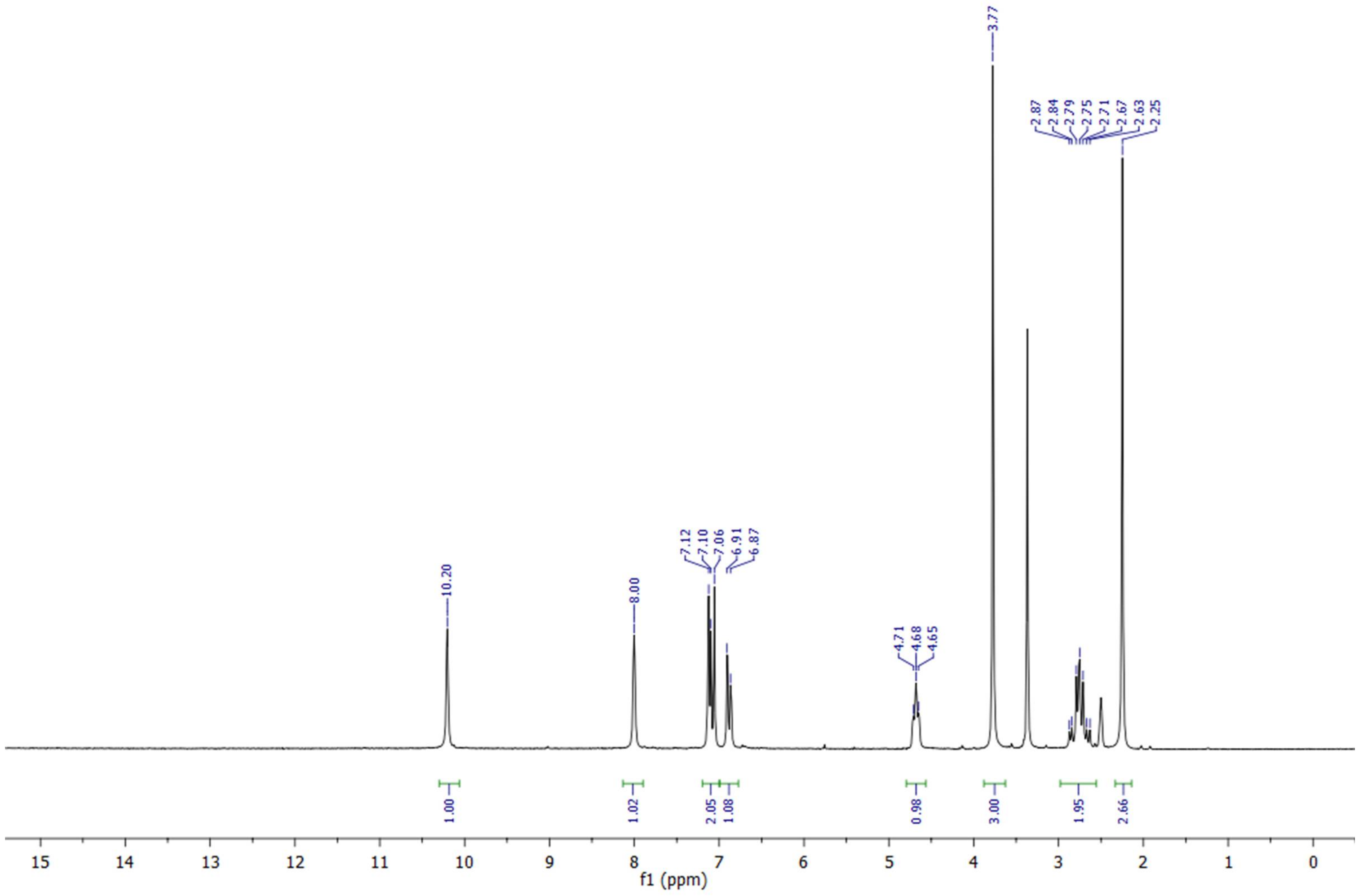

Figure S37. ^{1}H NMR spectrum of **2s**

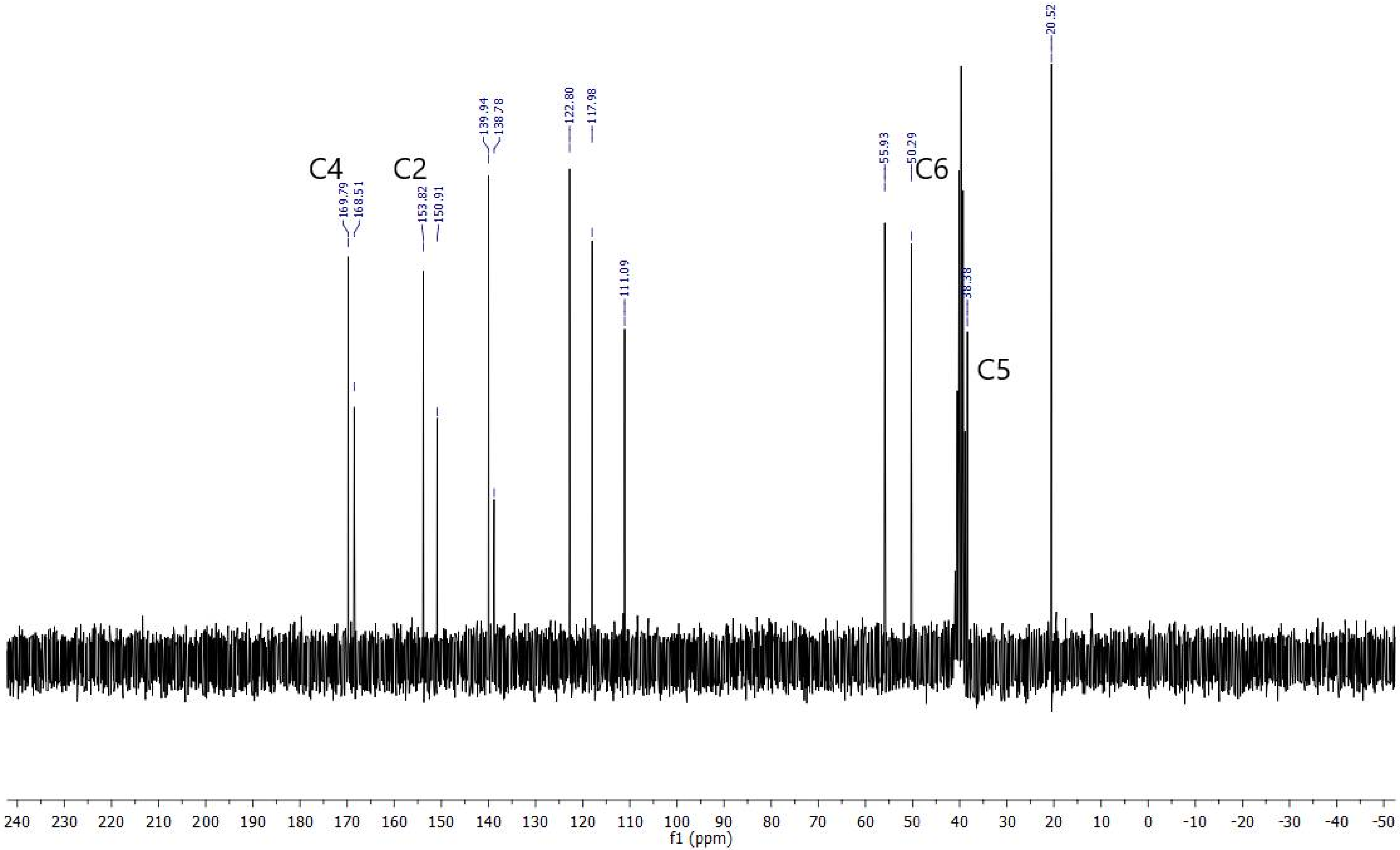

Figure S38. ^{13}C NMR spectrum of **2s**
